# Characterizing dislocation loops in irradiated polycrystalline Zr alloys by X-ray line profile analysis of powder diffraction patterns with satellites

**DOI:** 10.1107/S1600576721002673

**Published:** 2021-05-25

**Authors:** Tamás Ungár, Gábor Ribárik, Matthew Topping, Rebecca M. A. Jones, Xiao Dan Xu, Rory Hulse, Allan Harte, Géza Tichy, Christopher P. Race, Philipp Frankel, Michael Preuss

**Affiliations:** a Materials Performance Centre, University of Manchester, Manchester M13 9PL, United Kingdom; b Department of Materials Physics, Eötvös University, PO Box 32, Budapest H-1518, Hungary

**Keywords:** X-ray line profile analysis, small dislocation loops, total dislocation density of loops, dipole character of small loops, satellite peaks, types of dislocation loop, scattering by irradiation-induced dislocation loops

## Abstract

This work extends the convolutional multiple whole profile (CMWP) line profile analysis (LPA) procedure to determine the total dislocation density and character of irradiation-induced dislocation loops in commercial polycrystalline Zr specimens. Zr alloys are widely used in the nuclear industry as fuel cladding materials in which irradiation-induced point defects evolve into dislocation loops. LPA has long been established as a powerful tool to determine the density and nature of lattice defects in plastically deformed materials. The CMWP LPA procedure is based on the Krivoglaz–Wilkens theory in which the dislocation structure is characterized by the total dislocation density ρ and the dislocation arrangement parameter *M*. In commercial Zr alloys irradiation-induced dislocation loops broaden the peak profiles, mainly in the tail regions, and occasionally generate small satellites next to the Bragg peaks. In this work, two challenges in powder diffraction patterns of irradiated Zr alloys are solved: (i) determination of the *M* values from the long tail regions of peaks has been made unequivocal and (ii) satellites have been fitted separately, using physically well established principles, in order to exclude them from the dislocation determination process. Referring to the theory of heterogeneous dislocation distributions, determination of the total dislocation density from the main peaks free of satellites has been justified. The dislocation loop structure has been characterized by the total dislocation density of loops and the *M* parameter correlated to the dipole character of dislocation loops. The extended CMWP procedure is applied to determine the total dislocation density, the dipole character of dislocation loops, and the fractions of 〈*a*〉- and 〈*c*〉-type loops in proton- or neutron-irradiated polycrystalline Zr alloys used in the nuclear energy industry.

## Abstract 2

Satellites generated by small dislocation loops in polycrystalline proton- or neutron-irradiated Zr alloys are characterized by X-ray line profile analysis. The convolutional multiple whole-profile procedure has been extended to characterize irradiation-induced satellites around fundamental Bragg peaks.

## 1. Introduction   

In plastically deformed crystalline materials, dislocation densities, crystallite sizes and planar defect densities determined by transmission electron microscopy (TEM) and X-ray line profile analysis (LPA) reveal excellent correlation. In tensile deformed copper single crystals, dislocation densities have been determined by etch-pitting (Van Drunen & Saimoto, 1971 ▸), TEM (Essmann, 1965 ▸, 1966 ▸; Goettler, 1973 ▸; Ambrosi *et al.*, 1974 ▸, 1980 ▸) and X-ray LPA (Wilkens & Eckert, 1964 ▸; Wilkens & Bargouth, 1968 ▸; Wilkens, 1976 ▸; Ungár *et al.*, 1984 ▸; Mughrabi *et al.*, 1986 ▸). The dislocation density values provided by the three different methods were in very good agreement within experimental accuracy at the same plastic strain values. The crystallite size determined by X-ray LPA was critically scrutinized by Rebuffi *et al.* (2016 ▸). LPA was done by the whole powder pattern modelling (WPPM) method (Scardi & Leoni, 2002 ▸). The agreement between TEM and LPA results proved to be excellent, again within experimental accuracy. Planar defect densities were determined by TEM and X-ray LPA in sintered diamond–SiC nanocomposites (Gubicza *et al.*, 2007 ▸; Balogh *et al.*, 2008 ▸), providing very good correlation between the results of the two methods.

Although X-ray LPA has proved to be an excellent and effective complementary tool to TEM in determining dislocation densities and arrangements, crystallite sizes, and planar defect densities in crystalline materials with very different structures (Warren, 1959 ▸; Wilkens, 1970 ▸; Ungár *et al.*, 1999 ▸; Borbély & Groma, 2001 ▸; Scardi & Leoni, 2002 ▸; Ribárik & Ungár, 2010 ▸), some genuine problems and apparent discrepancies arise in data for irradiated materials such as irradiated zirconium alloys (Griffiths *et al.*, 1992 ▸, 2002 ▸; Griffiths, 2008 ▸; Balogh *et al.*, 2012 ▸, 2016 ▸, 2018 ▸; Harte *et al.*, 2015 ▸; Seymour *et al.*, 2017 ▸; Topping *et al.*, 2018 ▸, 2019 ▸):

(i) The dislocation densities determined by LPA are often considerably larger than those obtained by TEM (Seymour *et al.*, 2017 ▸; Topping *et al.*, 2019 ▸).

(ii) Satellites can appear in the tail regions of the peak profiles of powder diffraction patterns, making it difficult to use the conventional whole-profile fitting procedures (Harte *et al.*, 2015 ▸; Seymour *et al.*, 2017 ▸; Topping *et al.*, 2018 ▸, 2019 ▸).

Zirconium alloys are indispensable in the nuclear industry as fuel cladding materials for water-cooled reactors due to their low thermal neutron absorption cross section, acceptable mechanical properties and good corrosion resistance. Because of the extreme environment of concomitant fast neutron irradiation, high temperature and corrosive cooling media, zirconium cladding exhibits dimensional instabilities. This is caused by the cumulative effect of irradiation-induced growth (IIG), irradiation-enhanced creep, hydrogen pick up and hardening/embrittlement (Carpenter *et al.*, 1988 ▸; Onimus & Béchade, 2012 ▸; Adamson *et al.*, 2019 ▸). Neutron irradiation triggers cascades of vacancy and interstitial pairs (Carpenter *et al.*, 1988 ▸; Onimus & Béchade, 2012 ▸; Adamson *et al.*, 2019 ▸; Holt, 1988 ▸; Ziegler *et al.*, 2010 ▸). These point defects cluster to form vacancy- or interstitial-type dislocation loops, initially on the prismatic planes of Zr (Carpenter & Northwood, 1975 ▸; Jostsons *et al.*, 1977 ▸; Woo, 1998 ▸; Adamson, 2000 ▸; Varvenne *et al.*, 2014 ▸; 2016 ▸). Zr cladding tubes typically have a strong texture with split basal poles in the radial/transverse direction (Holt, 1988 ▸), resulting in growth in the axial and shrinkage in the radial direction (Carpenter *et al.*, 1988 ▸; Onimus & Béchade, 2012 ▸). During the first phase of IIG, dislocation loops, known as 〈*a*〉 loops, form on first- or second-order prismatic planes with 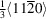 Burgers vectors (Kelly & Blake, 1973 ▸; Jostsons *et al.*, 1977 ▸; Northwood *et al.*, 1979 ▸; Griffiths, 1988 ▸; Lemaignan, 2012 ▸; Boyne *et al.*, 2013 ▸). They can be of either vacancy or interstitial type (Kelly & Blake, 1973 ▸; Griffiths *et al.*, 1983 ▸). At a later stage, 〈*c*〉 loops appear on basal planes with 〈*c*〉-type Burgers vectors (Jostsons *et al.*, 1977 ▸; Carpenter *et al.*, 1988 ▸; Holt, 1988 ▸). In a fully recrystallized material, TEM shows, on the one hand, the increase in the total dislocation density in the initial IIG phase (Williams *et al.*, 1984 ▸) and, on the other hand, the saturation of the number density of 〈*a*〉 loops during the steady-state growth phase (Carpenter *et al.*, 1988 ▸; Griffiths, 1988 ▸). In a later phase of service, the IIG rate increases and becomes the accelerated or ‘breakaway’ growth phase (Rogerson & Murgatroyd, 1983 ▸; Griffiths, 1988 ▸; Rogerson, 1988 ▸; Griffiths *et al.*, 1989 ▸). With increased dose large faulted vacancy-type dislocation loops, known as 〈*c*〉 loops, are also observed to form on the basal plane with a Burgers vector of 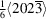 (Holt & Gilbert, 1986 ▸; Griffiths & Gilbert, 1987 ▸). In addition to IIG, the formation of dislocation loops leads to hardening and embrittlement of the material (Northwood *et al.*, 1979 ▸; Griffiths, 1988 ▸; Lemaignan, 2012 ▸). Though the vast majority of microstructure investigations during IIG have been based on TEM (Griffiths *et al.*, 1983 ▸; Carpenter *et al.*, 1988 ▸), X-ray and neutron LPA have also been applied to follow the IIG process (Griffiths *et al.*, 1992 ▸, 2002 ▸; Griffiths, 2008 ▸; Balogh *et al.*, 2012 ▸, 2016 ▸; Harte *et al.*, 2015 ▸; Seymour *et al.*, 2017 ▸; Topping *et al.*, 2019 ▸). Harte *et al.* (2015 ▸), Balogh *et al.* (2016 ▸), Seymour *et al.* (2017 ▸) and Topping *et al.* (2019 ▸) carried out systematic TEM and X-ray LPA analysis on different neutron- or proton-irradiated Zr alloy specimens.

Zr alloys are designed to improve and optimize all the alloys’ properties without altering their key characteristic, which is their low thermal neutron absorption cross section. First, we need to understand what happens to the microstructure during irradiation within various environments. Different electron microscopy methods provide a comprehensive qualitative and, to some extent, quantitative description of the microstructure in irradiated Zr alloys (Jostsons *et al.*, 1977 ▸; Griffiths *et al.*, 1983 ▸; Carpenter *et al.*, 1988 ▸). However, some fundamental features of the microstructure, such as the density of dislocation loops, strains related to dislocation loops and the quantitative ratios of different loop types, can be better characterized by X-ray diffraction experiments.

In the present work we extend the convolutional multiple whole profile (CMWP) LPA procedure to determine the total dislocation density ρ and the dislocation arrangement parameter *M* in neutron- or proton-irradiated commercial polycrystalline Zr alloys. Irradiation-induced dislocation loops usually have wide size distributions. Small loops have short-range strain fields with strong dipole character, producing long tail regions in diffraction peaks and occasionally small satellites next to the Bragg peaks. With increasing loop size, the range of strain fields increases with decreasing dipole character. Due to the wide size distributions of the loops, the complex strain fields generate global broadening both at the centre and in the tail regions of the peaks. The total dis­location density is obtained from the main diffraction peaks, so the small satellites are fitted separately in order to exclude them from the dislocation determination procedure. We show that the strained volume of small dislocation loops (SDLs) scales with the reciprocal loop size. Utilizing this property, we fitted the satellite peaks by profile functions of small scattering objects with a log-normal size distribution. The main peaks are evaluated using the two-parameter strain profile model of Wilkens (1970 ▸). We recognize that when very small loops are generated with large dislocation densities the effective outer cut-off radius, *R*_e_, can approach the lower length limit of the continuum approximation, where *R*_e_ is related to ρ and *M* as *M* = *R*_e_(ρ^1/2^). In order to avoid *R*_e_ becoming smaller than the lower length limit of the continuum approximation we introduce a user-specified hard limit, *R*_c_, for *R*_e_. With the introduction of *R*_c_ the extended CMWP procedure becomes robust to evaluate the total dislocation density of densely populated very small loops. The dislocation contrast factors play a crucial role in the CMWP procedure. Balogh *et al.* (2016 ▸) evaluated contrast factors for irradiation-induced dislocation loops in Zr. Using these contrast factors we develop a simple and straightforward method to determine the dislocation density fractions pertaining to 〈*a*〉- and 〈*c*〉-type dislocation loops.

Considering the temperature dependence of loop density and size in proton-irradiated Zircaloy-2 specimens, we discuss and interpret the apparently controversial results of Seymour *et al.* (2017 ▸) that, in neutron-irradiated Zircaloy-2 specimens stemming from operating nuclear reactors, while the TEM-counted dislocation densities are smaller in the channel than in the cladding materials, the CMWP-determined values show the opposite, *i.e.* are larger in the channel than in the cladding materials. The extension of the CMWP procedure is implemented in a general manner, making it applicable to the evaluation of various powder diffraction patterns revealing satellites around the Bragg reflections.

## 2. Experimental   

### 2.1. Materials   

For the present work, samples of proton- and neutron-irradiated Zircaloy-2 with slightly different chemical compositions were available. The proton-irradiated Zircaloy-2 had a composition of Zr, 1.5 Sn, 0.1 Fe, 0.1 Cr, 0.06 Ni (Hallstadius *et al.*, 2012 ▸) while the neutron-irradiated Zircaloy-2 had a nominal composition of Zr, 1.34–1.35 Sn, 0.17–0.18 Fe, 0.11 Cr, 0.05–0.07 Ni, all in wt%. Three proton-irradiated specimens were prepared from recrystallized Zircaloy-2. For reference purposes one non-irradiated specimen of the same material was also tested. The specimens were proton-irradiated either at the Michigan Ion Beam Laboratory at the University of Michigan, USA, using a 1.7 MeV Tandetron accelerator (Was & Rotberg, 1989 ▸), or at the Dalton Cumbrian Facility (DCF) of the Dalton Nuclear Institute, UK (Topping *et al.*, 2019 ▸). The cold-rolled and recrystallized bars were irradiated along the normal direction using 2 MeV protons at a current of ∼20 µA cm^−2^. The samples were irradiated to the level of 2.3 dpa (displacement per atom) at 280, 350 and 450°C at a dose rate of about 1.3 × 10^−5^ dpa s^−1^. Damage rates are for 40% depth of the Bragg peak as calculated using the *Stopping and Range of Ions in Matter* (*SRIM*) software package, assuming pure Zr with a displacement energy of 40 eV (Ziegler *et al.*, 2010 ▸). More details about the proton-irradiated specimens are given by Topping *et al.* (2019 ▸).

The provision of neutron-irradiated Zircaloy-2 specimens from commercial reactors was in the form of electro-polished TEM foils provided by Westinghouse Electric Company and Studsvik Nuclear AB (Valizadeh *et al.*, 2011 ▸). The neutron fluence, number of cycles, approximate core elevation of the Zr rods and rod-growth values are given in Table 1 of Seymour *et al.* (2017 ▸). Four specimens were taken from cladding and two from channel materials. The operation temperature of the cladding materials was 350 ± 10°C, whereas the channel materials were neutron-irradiated at a lower temperature of approximately 300 ± 10°C (Valizadeh *et al.*, 2011 ▸). More details about the neutron-irradiated specimens are given by Seymour *et al.* (2017 ▸).

### 2.2. X-ray diffraction experiments   

X-ray diffraction patterns were collected on the I11 high-resolution powder diffraction synchrotron beamline at the Diamond Light Source, UK (Thompson *et al.*, 2009 ▸). The monochromatic and parallel X-ray beam was operated at about 15 keV with a wavelength of λ = 0.08259 nm. The analyser crystals in the diffracted beam and the low-noise detectors ensured low background and good peak-to-background ratios (Tartoni *et al.*, 2008 ▸). The step size was Δ2θ = 0.001°, allowing at least 15 data points above the FWHM, even in the narrowest peaks. The neutron-irradiated TEM foil specimens were measured in transmission mode (Seymour *et al.*, 2017 ▸). The instrumental effect was determined using a silicon standard specimen. Proton irradiation results in a damage profile unevenly confined to a near-surface region with a high Bragg peak around a depth of about 30 µm, as shown in Fig. 1 of Topping *et al.* (2019 ▸). In order to ensure that the majority of the X-ray signal was obtained from the plateau region of the damage profile at around 5 µm, sampling approximately the same depth as in the TEM investigations, the incidence angle for the proton-irradiated specimens was held constant at about 5°, carrying out the measurements in reflection mode.

### 2.3. Principles of X-ray line profile analysis   

The X-ray diffraction patterns were evaluated by the CMWP procedure (Ribárik, 2008 ▸; Ribárik & Ungár, 2010 ▸; Ribárik *et al.*, 2019 ▸). The principles of the method are based on physically well established profile functions accounting for broadening by crystallite size, heterogeneous strain and planar defects (Warren, 1959 ▸; Krivoglaz & Rjaboshapka, 1963 ▸; Wilkens, 1970 ▸; Groma, 1998 ▸; Ungár *et al.*, 1999 ▸; Scardi & Leoni, 2002 ▸; Ribárik & Ungár, 2010 ▸; Zilahi *et al.*, 2015 ▸; Ribárik *et al.*, 2019 ▸). Profile functions corresponding to the different physical effects are superimposed by convolution (Warren, 1959 ▸),
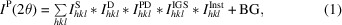
where *I* ^P^(2θ) is the physically established diffraction pattern, 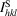, 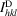 and 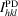 are the physically based theoretical size, strain (distortion) and planar defect profile functions, respectively, 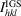 is the profile function related to elastic intergranular strains, 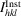 is the measured instrumental profile, and BG is the background. Thermal diffuse scattering (TDS) has been subtracted and included in the BG, similarly to the approach used by Li *et al.* (2009 ▸). However, in the present case the TDS was not interpreted. The size profile is calculated by taking into account the shape and size distribution of coherently scattering domains (Bertaut, 1950 ▸). Assuming a log–normal size distribution, the Fourier transform of the size profile of spherical crystallites can be written as (Ungár *et al.*, 2001 ▸; Ribárik, 2008 ▸)
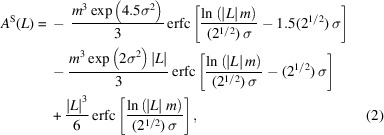
where *L* is the Fourier variable, *m* and σ are the median and variance, respectively, of the log–normal size distribution density function, and erfc is the complementary error function. Given *m* and σ, the arithmetic and area- and volume-weighted mean crystallite diameters can be calculated (Hinds, 1982 ▸):
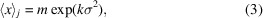
where *k* = 0.5, 2.5 and 3.5 for the arithmetic and area- and volume-weighted means, respectively, and *j* stands for these averages. The Fourier transform of the strain profile is (Warren, 1959 ▸)
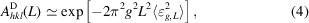
where *g* is the absolute value of the diffraction vector, *L* is the Fourier variable and 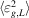 is the mean-square strain (m.s.s.). In crystals containing dislocations the m.s.s. can be given as (Wilkens, 1970 ▸)
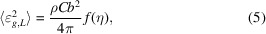
where ρ and *b* are the density and the length of the Burgers vector of dislocations, respectively, *C* is the dislocation contrast factor, η = *L*/*R*_e_, and *R*_e_ is the effective outer cut-off radius of dis­locations. *R*_e_ has the same physical meaning in line broadening as in the elastic stored energy of dislocations (Nabarro, 1952 ▸; Wilkens, 1969 ▸). The short- or long-range character and the arrangement of dis­locations can be characterized by *R*_e_ relative to the average dislocation distance *d* = 1/(ρ^1/2^). When the dislocation arrangement is the same, whereas the dislocation density varies, the value of *R*_e_ will depend on the actual value of ρ. Therefore, the arrangement of dislocations can be better characterized by the ρ-independent dimensionless number *M* = *R*_e_(ρ^1/2^). For strongly correlated dislocation arrangements with short-range strain fields *M* ≤ 1, whereas for random dislocation arrangements with long-range strain fields *M* ≫ 1. In crystal or reciprocal space the length scales are reciprocal. Therefore, diffraction from short- or long-range strain fields extends to long or short distances from the exact Bragg positions. This means that short- or long-range strain fields produce long or short tails in diffraction peaks, related to *M* ≤ 1 or *M* ≫ 1, respectively.

**Figure 1:**
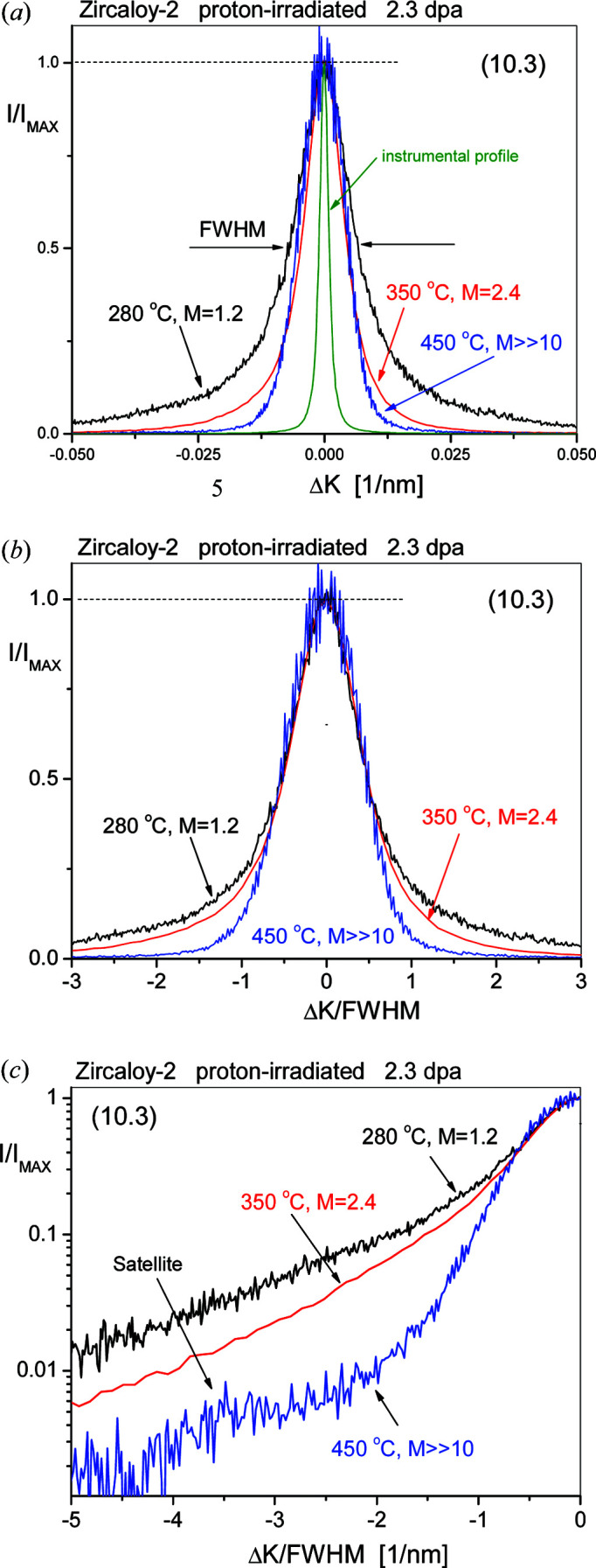
(*a*) Peak profiles of the (10.3) reflections of Zircaloy-2 proton-irradiated to a level of 2.3 dpa at 280, 350 and 450°C. (The smaller noise in the 350°C profile is due to the longer data collection time in the diffraction experiment.) The profiles are normalized to the peak maxima. The green line is the narrow instrumental profile. (*b*) The same profiles as in (*a*), normalized to both the peak maxima and the FWHM. (*c*) One half of the peaks in (*b*) with the normalized intensities on a logarithmic scale. Note that the background has been subtracted from all the diffraction peaks shown in panels (*a*), (*b*) and (*c*).

**Figure 2:**
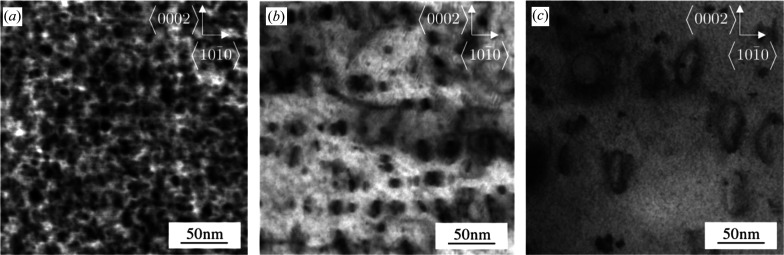
TEM micrographs of 〈*a*〉 loops induced by proton irradiation at (*a*) 280°C, (*b*) 350°C and (*c*) 450°C.

SDLs have strong dipole character and their strain fields are of short-range character (Kroupa, 1966 ▸). With growing loop size the strain field gradually becomes of long-range character. Small or large dislocation loops and narrow or wide ± sign dislocation dipoles have similar short- or long-range strain fields. Therefore, when either dislocation loops or dislocation dipoles are the major defect type, the tail regions of diffraction peaks in powder diffraction patterns can be characterized by the *M* parameter. The qualitative correlation between peak tails in powder diffraction patterns and loop size or density is shown in Figs. 1 ▸ and 2 ▸. Fig. 1 ▸ shows profiles of the (10.3) reflection of Zircaloy-2 proton-irradiated to 2.3 dpa at 280, 350 and 450°C. Fig. 1 ▸(*a*) shows that the FWHMs vary within a small range with irradiation temperature, whereas the tail regions show a large variation in broadening. In Fig. 1 ▸(*b*) the profiles are normalized to both the peak maxima and FWHMs. The tail regions of the peaks become longer with decreasing irradiation temperatures. The longer tail regions are more pronounced in Fig. 1 ▸(*c*) where only half of each profile is shown. The TEM micrographs in Fig. 2 ▸ show the 〈*a*〉 loops in the same three proton-irradiated Zircaloy-2 specimens. In Fig. 2 ▸(*a*), at 280°C irradiation temperature, the loops are small with large density. At higher irradiation temperatures, *i.e.* in Figs. 2 ▸(*b*) and 2 ▸(*c*), the loops become larger with smaller number densities.

Although Wilkens (1970 ▸) developed the *f*(η) strain function based on straight parallel screw dislocations, several authors have shown that the two-parameter model with ρ and *M* is far more general and is also applicable for curved or edge dislocations (Groma, 1998 ▸; Kamminga & Delhez, 2000 ▸; Groma & Monnet, 2002 ▸; Csikor & Groma, 2004 ▸; Ribárik, 2008 ▸). Csikor & Groma (2004 ▸) extended the *f*(η) function to describe the width of dipoles by including a third parameter. The three-parameter strain function, *f*_Cs-G_(η), is implemented in the CMWP procedure (Ribárik, 2008 ▸), and it can be shown that, for a given dipole width, *f*(η) and *f*_Cs-G_(η) are identical (Ribárik, 2008 ▸). Although *f*_Cs-G_(η) is more general than *f*(η), experience has shown that when the dipole-width distribution is wide, as in the case of our specimens, the dipole-width evaluation is rather uncertain. Therefore, in the present work, in all CMWP evaluations we use the two-parameter strain function.

**Figure 3:**
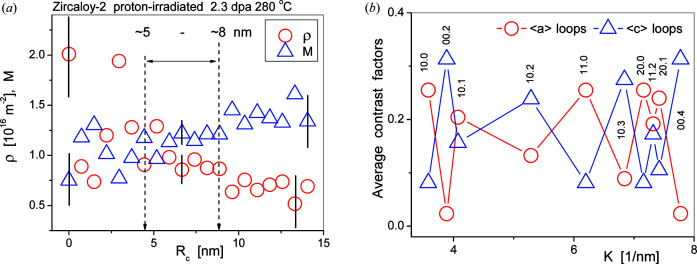
(*a*) The values of ρ (open red circles) and *M* (open blue up-triangles) versus *R*_c_ defined in equation (6). The vertical black lines indicate typical errors within different ranges of *R*_c_. (*b*) Average contrast factors of 〈*a*〉 loops (open red circles) and 〈*c*〉 loops (open blue up-triangles) as determined by Balogh *et al.* (2016 ▸).

Irradiation-induced dislocation loops have a wide size distribution which can range between about 0.5 and 100 nm (Larson & Young, 1987 ▸; Sand *et al.*, 2013 ▸; Mason *et al.*, 2014 ▸) and can have large densities (Seymour *et al.*, 2017 ▸; Topping *et al.*, 2019 ▸). Under these circumstances *R*_e_ = *M*/(ρ^1/2^) might approach the lower limit in the continuum approximation (Wagner & Liu, 2003 ▸; Seif *et al.*, 2015 ▸). In order to avoid this kind of singularity we introduced a hard lower limit, *R*_c_, into the strain function,
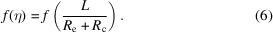



When *R*_e_ ≫ *R*_c_ the renormalized Wilkens function becomes the original one, while the denominator in *f*(η) can never go below *R*_c_. With the introduction of *R*_c_ the CMWP procedure has become robust, even when the dislocation loops have a very strong dipole character along with a very high loop density. *R*_c_ has been introduced into CMWP as a user-defined parameter. A systematic investigation of the effect of *R*_c_ on ρ and *M* is shown in Fig. 3 ▸(*a*). The figure shows that the scattering of the ρ and *M* values is smallest and the stability is the best when the value of *R*_c_ is between 5 and 8 nm.

The contrast factor *C* accounts for strain anisotropy and can be calculated theoretically for any relative orientation between the Burgers vectors **b** and line vectors **l** of dis­locations, and of diffraction vectors **g**, taking into account the elastic anisotropy of crystals (Hirsch *et al.*, 1965 ▸; Wilkens, 1970 ▸; Krivoglaz, 1996 ▸; Borbély *et al.*, 2003 ▸; Leoni *et al.*, 2007 ▸). In a polycrystal *C* can be averaged over the permutations of *hkl*s. Ungár & Tichy (1999 ▸) showed that the *hkl* dependence of the average contrast factor 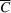 is a linear function of the fourth-order invariant of *hkl*s. For hexagonal close-packed (h.c.p.) crystals the average contrast factor is (Dragomir & Ungár, 2002*a* ▸)
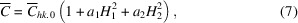
where
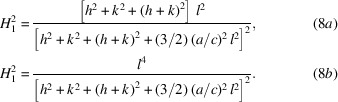
Here, *a* and *c* are the lattice constants of the h.c.p. crystal. In the CMWP procedure for h.c.p. materials, the *hkl* dependence of 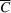 is accounted for by the *a*_1_ and *a*_2_ parameters. The average contrast factors of 〈*a*〉 and 〈*c*〉 loops for the first ten reflections of Zr, as calculated by Balogh *et al.* (2016 ▸), are shown in Fig. 3 ▸(*b*). The contrast factors of the two different loop types are almost in anti-coincidence. The measured and calculated diffraction patterns are matched by combining the Marquart–Levenberg (ML) nonlinear least-squares procedure and a recently implemented Monte Carlo (MC) statistical algorithm (Ribárik *et al.*, 2019 ▸, 2020 ▸). The two procedures are applied iteratively in order to obtain the global minimum of the weighted sum of squared residuals between the measured and calculated patterns and the global optimum of the physical parameters.

### 2.4. Satellites around the fundamental Bragg reflections produced by irradiation-induced dislocation loops   

**Figure 4:**
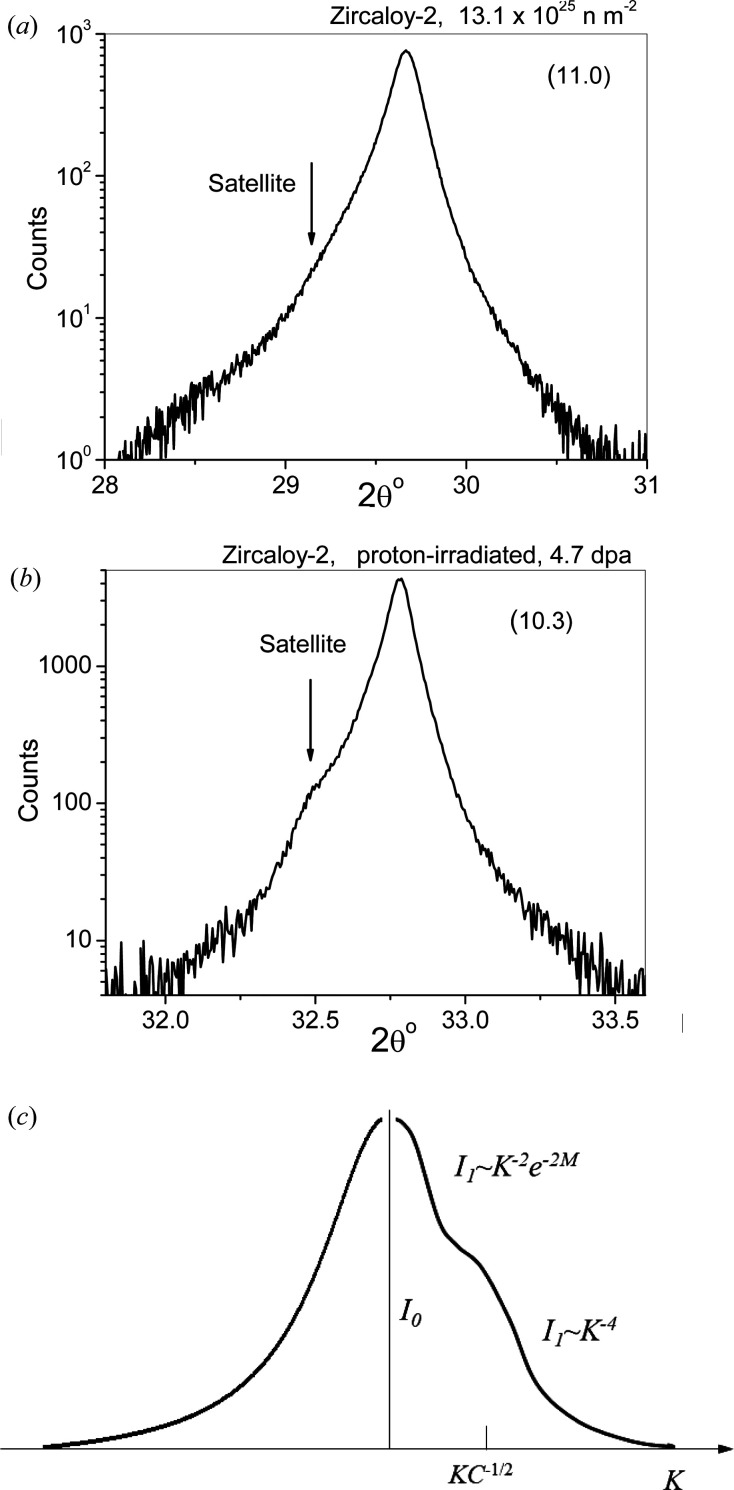
(*a*) The peak profile of the (11.0) reflection of a Zircaloy-2 specimen neutron-irradiated to a fluence of 13.1 × 10^25^ n m^−2^ on a logarithmic intensity scale. (*b*) The peak profile of the (10.3) reflection of a Zircaloy-2 specimen proton-irradiated to a level of 4.7 dpa at 350°C on a logarithmic intensity scale. The logarithmic intensity scale is used in order to visualize the satellite peaks in the low-intensity regions of the profiles. (*c*) Redrawn from Fig. 40.18(*b*) of Krivoglaz (1996 ▸), where *I*_0_ denotes the peak of the undistorted perfect crystal, exp(−2*M*) is the Debye–Waller factor, *C* is the strength of the defects and *I*_1_ is the defect-affected intensity. Note that the satellites in panels (*a*) and (*b*) are related to vacancy-type loops, whereas the ‘hump’ in panel (*c*) corresponds to interstitial-type defects.

Neutron or proton irradiation in Zr alloys can produce satellites around the Bragg peaks in X-ray powder diffraction patterns (Seymour *et al.*, 2017 ▸). Two typical powder diffraction profiles, one of neutron- and one of proton-irradiated Zircaloy-2, are shown in Figs. 4 ▸(*a*) and 4 ▸(*b*), respectively. Note the logarithmic intensity scale for better observation of the satellites. The effect of dislocation loops on peak profiles is shown schematically in the book by Krivoglaz (1996 ▸) in Fig. 40.18. The four figures from (*a*) to (*d*) are scaled with increasing ‘strength’, *C*, of ‘defects’, where *C* = Δ*V*/*V* is the relative volume change around defects. There is a striking similarity between the peak shape of the profile in Fig. 4 ▸(*b*) and the schematic peak shape in Fig. 40.18(*b*) of Krivoglaz (1996 ▸), redrawn after Krivoglaz as Fig. 4 ▸(*c*). Note that the ‘hump’ in Fig. 4 ▸(*b*) is related to vacancy loops, whereas that in Fig. 40.18(*b*) [and Fig. 4 ▸(*c*)] is related to interstitial-type defects. The exp(−2*M*) factor is the Debye–Waller factor of peak intensity.

In strongly distorted polycrystalline powder patterns the peaks related to the undistorted perfect crystal, denoted *I*_0_ in Fig. 40.18(*b*) and Fig. 4 ▸(*c*), are completely lost, and therefore in the CMWP procedure the peak intensities become irrelevant and the Debye–Waller factor is not used. As long as the strain fields around the loops are well separated and the loops are randomly distributed, the generated scattering can be calculated without taking into account interaction between strain fields. In such cases the diffuse scattering around the Bragg peaks can be described by the sum of scattering generated by individual clusters or small loops, as in the single-defect approximation (SDA) developed by Dederichs (1971 ▸), Larson & Young (1987 ▸) and Ehrhart & Averback (1989 ▸). Loop properties and their effect on peak profiles are best understood by the SDA. Therefore, we briefly summarize the basic features of the SDA.

In the SDA, when non-interacting SDLs are present the scattering amplitude can be written as (Dederichs, 1971 ▸)
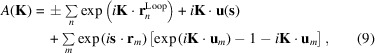
where **K** = **g** + **s**, **g** is the exact Bragg position of the fundamental *hkl* reflection and **s** is the ‘deviation vector’ (a term used in electron microscopy) in the vicinity of **g**. In the case of vacancy loops, 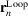 is the position vector of those atoms which were removed from the crystal, and in this case we use a positive sign in front of the first sum. For interstitial loops, 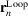 is the position vector of the atoms added to the crystal, and in this case a negative sign is valid in front of the first sum. We note that for slip-type dislocation loops no atoms need to be removed or added. **u** *_m_* is the displacement of the *m*th atom relative to the average crystal. The **u** *_m_* vector is extended continuously throughout the entire crystal, where **u**(**s**) is the Fourier integral of this function.

**Figure 5:**
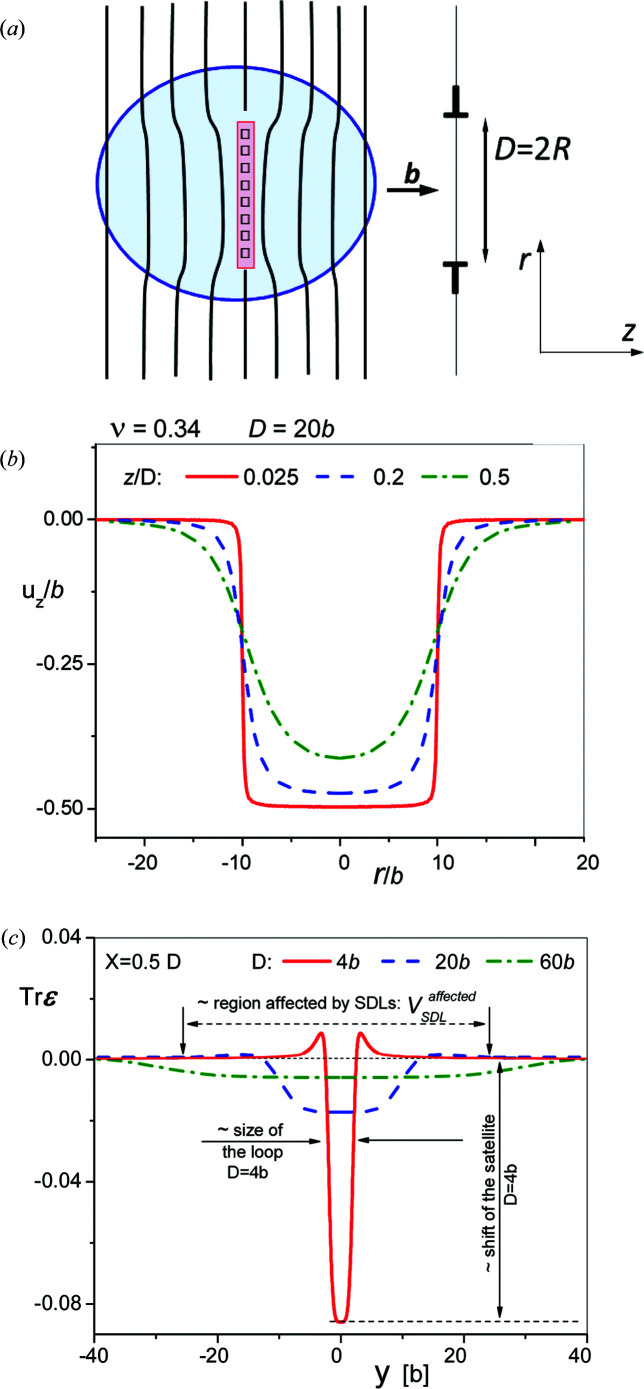
(*a*) A schematic image of a prismatic vacancy-type SDL, where **b** is the Burgers vector, *D* is the loop diameter, the ‘T’s indicate edge dislocation and the polar coordinates follow the notation of Kroupa (1960 ▸). Open squares indicate vacancies, vertical black lines indicate lattice planes, and the light-red and light-blue areas show the volume and the affected regions of the loop, respectively. (*b*) The displacement field of a vacancy-type prismatic SDL of 20*b* diameter, *D* = 20*b*, at different heights, *z*/*D*, from the plane of the loop. (*c*) The trace of the strain tensor, 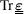, of vacancy-type prismatic SDLs of three different diameters: *D* = 4*b* (red line), 20*b* (dashed blue line) and 60*b* (dashed–dotted green line). Both the displacements and the 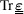 values were calculated using the equations of Kroupa (1960 ▸) with Poisson’s number for Zr (Weck *et al.*, 2015 ▸).

The first, direct, term in equation (9) gives only a small contribution to *A*(**K**) because the volume of vacancies or interstitials, *b*π(*D*/2)^2^, forming the loop is small compared with the volume affected by the strain fields around the SDLs (*D* is the diameter of the loops). A schematic drawing of a small prismatic vacancy-type loop with the volume comprising the direct term (light-red shaded region) and the volume affected by the strain field (light-blue shaded region) is shown in Fig. 5 ▸(*a*).

The second, first-order distortion scattering (FODS), term is centred around the *hkl* Bragg position since **u**(**s**) is the Fourier transform of the real **u**(**r**) displacement field. The related scattered intensity, *I*_FODS_(**s**) = 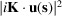, is discussed in more detail below. The third, higher-order, term is generated from the volume affected by the strain field of the loops. [The names used for the three terms in equation (9) are taken following Iida *et al.* (1988 ▸).]

Kroupa (1960 ▸) calculated the stress, deformation and deformation energy of a circular prismatic dislocation loop employing the symmetry of the problem with respect to the plane of the loop at *z* = 0 [see Fig. 5 ▸(*a*)]. The displacement field components are given in equation (16) of Kroupa (1960 ▸). The *u_z_*(*r*) component has been calculated using equations given by Kroupa (1960 ▸) [equation (16) by numerical integration of the Bessel functions in equation (14)] and is shown at three different heights above the plane of the loop in Fig. 5 ▸(*b*). Applying Hook’s law, the trace of the strain tensor can be expressed as:
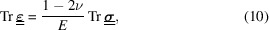
where 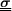 is the stress tensor, ν is the Poisson number and *E* is Young’s modulus. Using equations (14) and (15) of Kroupa (1960 ▸) and equation (10) above, 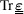 can be written as
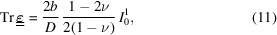
where 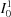 is the integral of the Bessel functions in equation (14) of Kroupa (1960 ▸). Fig. 5 ▸(*c*) shows 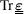, which is the relative volume change Δ*V*/*V*, along the *r* direction for three different loop diameters: *D* = 4*b*, *D* = 20*b* and *D* = 60*b*.

On the basis of equation (10) and Fig. 5 ▸(*c*), the qualitative features of satellites can be summarized as follows:

(i) Vacancy- or interstitial-type SDLs produce satellites on the smaller or larger angle sides of the fundamental Bragg reflections due to the dilated or compressed regions around the loops, respectively.

(ii) Satellite scattering is given by the sum of the direct and higher-order terms in equation (9).

(iii) The shift of satellites is proportional to the local volume changes inside the loops, as indicated in Fig. 5 ▸(*c*) for the *D* = 4*b* wide loop at *r* = 0.5*D*.

(iv) With increasing loop size, the volume change inside the loops tends to zero and the loops have the same effect on line broadening as ordinary lattice dislocations.

(v) Equation (11) and Fig. 5 ▸(*c*) indicate a strict correlation between the shifts and the diameters of the loops: 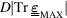 = Const. [In the case of Zr with a Poisson constant ν = 0.34 (Weck *et al.*, 2015 ▸), 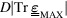 ≃ 0.34.]

**Figure 6:**
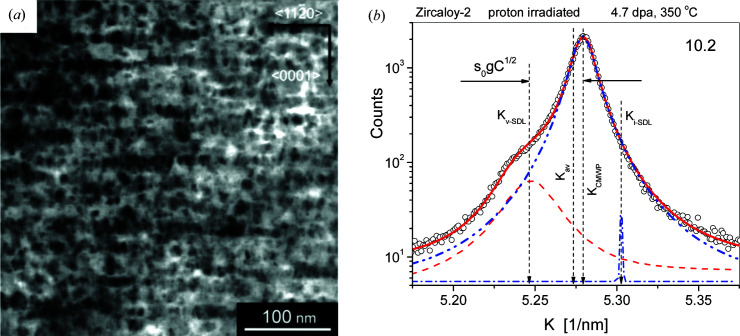
(*a*) A TEM micrograph of 〈*a*〉-type dislocation loops in Zircaloy-2 proton-irradiated to 4.7 dpa at 350°C. [Adapted with permission from Harte *et al.* (2017 ▸) under a Creative Commons CC-BY License, https://creativecommons.org/licenses/by/4.0/.] (*b*) The peak profile of the 10.2 reflection of a Zircaloy-2 specimen proton-irradiated to 4.7 dpa at 350°C on a logarithmic intensity scale. The open circles are the measured data, the solid red line is the CMWP-calculated profile, and the blue dashed–double-dotted line is the CMWP-calculated physical profile without the satellite intensities and without the instrumental effect. (Note that the background is still present in the plotted curves.) The red dashed and the blue dashed–dotted lines are the satellite profiles of the vacancy- and interstitial-type 〈*a*〉 loops, respectively. The shift in the vacancy-loop satellite relative to the main peak is indicated as Δ*K*_v-SDL_ = *s*_0_ *gC* ^1/2^. The *s*_0_ shift parameter and the intensities of the satellite peaks are global values for the entire pattern.

The SDA was successfully used to evaluate the experiments of Larson & Young (1987 ▸) and Ehrhart & Averback (1989 ▸), where the specimens were high-purity defect-free single crystals before irradiation. Diffuse X-ray scattering was determined by rocking-curve experiments in high-resolution three-crystal diffractometers. The specimens used in the present work, however, were commercial polycrystalline Zr alloys with an average grain size of about 10 µm. Our specimens were proton- or neutron-irradiated to substantially higher dose levels at elevated temperatures between 280 and 450°C. The TEM micrograph in Fig. 6 ▸(*a*) of a Zircaloy-2 specimen proton-irradiated to 4.7 dpa at 350°C (Harte *et al.*, 2017 ▸) shows the alignment of 〈*a*〉 loops along the 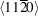 basal direction, indicating a strong interaction between the strain fields of the loops. The neutron-irradiated specimens used in the present work were obtained from operating nuclear reactors with a lowest irradiation dose level of 8.7 × 10^25^ n m^−2^, irradiated around the operation temperatures of the nuclear power stations. This means that, in the specimens used in the present work, the dose levels are orders of magnitude larger and the irradiation temperatures substantially higher than in the specimens investigated by Larson & Young (1987 ▸) or Ehrhart & Averback (1989 ▸).

The TEM micrographs shown by Harte *et al.* (2017 ▸) [see also Fig. 6 ▸(*a*)] indicate that, in the present specimens, the strain fields around the loops interact strongly and the conditions for the applicability of the SDA do not hold. The peak profiles from our heavily distorted polycrystalline specimens are strongly broadened, often over-masking the satellites, as shown for example in Fig. 1 ▸(*a*). The FODS term in equation (9) is similar to Huang scattering as long as the loops are non-interacting and small. In this case scattering from the average lattice can be fully subtracted by the −1 in the third term, the small non-interacting loops have non-touching short-range strain fields and the SDA can be applied. However, when the loops have a wide size distribution and large densities, as in the present case, the strain fields overlap strongly and the FODS term gives line broadening, as in the case of ordinary lattice dislocations. In such cases the FODS term describes the strain-broadened main diffraction peaks and is no longer similar to Huang scattering.

Groma and co-workers (Groma *et al.*, 1988 ▸; Groma, 1998 ▸) have shown that asymmetric peak profiles produced by polarized dislocation dipoles in dislocation cell structures of tensile deformed Cu specimens can be described by the complex Fourier transform
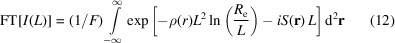
(with *L* < *R*_e_), where the first term within the square brackets is the logarithmic part in the *f*(η) strain function, ρ is the total dislocation density and *F* is related to the contrast of dis­locations. Equation (12) is valid for *L* values smaller than *R*_e_. The second, imaginary, part describes the polarization of the dislocation structure accounting for the asymmetry of the peaks. In the dislocation cell structure of tensile deformed Cu single crystals, the dislocation dipoles are aligned along the boundaries between cell walls and cell interiors, introducing compressive and tensile residual stresses in the cell-interior and cell-wall regions, respectively (Ungár *et al.*, 1984 ▸; Mughrabi *et al.*, 1986 ▸). The Burgers vectors of irradiation-induced 〈*a*〉 loops in Zr alloys are aligned along the basal directions, meaning that the dislocation dipoles related to these loops are polarized along the basal directions. In equation (9) the FODS term is decoupled from the terms of satellite scattering. In equation (12) the Fourier transform of the scattered intensity is decoupled into a real part containing the total dislocation density and an imaginary part describing the asymmetry of the peaks, related to the net polarization of dislocation dipoles. In equation (9) the FODS term with **u**(**s**) and in equation (12) the real part with ρ describe the same leading parts of peak profiles symmetrically centred around the fundamental Bragg positions. From this, we conclude that the total dislocation density related to SDLs can be obtained from the main diffraction peak free from satellites, using the two-parameter strain function *f*(η).

On the basis of the above analysis, satellites and the main diffraction peaks are fitted separately and the total dislocation density ρ and dipole character *M* are determined from the main peaks. Satellite property (v), in the list above, reveals a reciprocal correlation between the size and shift, *D* and 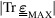, of satellites, indicating that both the size distribution and the shift in scattering of SDLs follow the same distribution function. We assume that the size distributions of SDLs can be given as log-normal distributions and we model the satellite profile functions 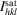 by the functional form described in equation (2), with the median *m* ^sat^ and variance σ^sat^ of the log-normal size-distribution parameters related to the satellites. In the extended CMWP procedure the satellite profiles are added to the main diffraction peaks, modifying equation (1) as
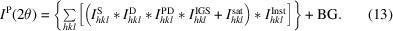
Four separate satellites on each Bragg peak are allowed, where the loop-related contrast factor parameters, the number of satellites to be fitted and the types of satellites, *i.e.* vacancy or interstitial type, can be edited by the user.

The shifts of satellites relative to the main peaks, 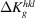, are *hkl* dependent because both the diffraction vector **g** and the contrast factors of the loops depend on *hkl*. The peak shifts, Δ*d*/*d*, scale with the square root of the stored energy, *W* ^1/2^ = (1/2*c_ij_*ɛ_*i*_ɛ_*j*_)^1/2^, where *c_ij_* are the elastic constants and ɛ_*i*_, ɛ_*j*_ are the elastic tensor components in the sextic formalism (Borbély & Driver, 2004 ▸). The dislocation contrast factors are linear functions of the elastic constants (Borbély *et al.*, 2003 ▸) and Δ*d*/*d* = −Δ*K*/*g*. With these considerations,
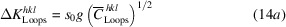
and
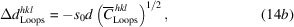
where *s*_0_ is an *hkl*-independent global shift parameter for all the Bragg peaks in the whole diffraction pattern. The shift notations are shown in Fig. 6 ▸(*b*) on the (10.2) reflection of a Zircaloy-2 specimen proton-irradiated to 4.7 dpa at 350°C. In equations (14*a*)[Disp-formula fd14a] and (14*b*)[Disp-formula fd14b], 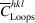 are the *hkl*-dependent average contrast factors for dislocation loops (Balogh *et al.*, 2016 ▸). They are shown for dislocation loops in Zr in Fig. 3 ▸(*b*). The intensities of the satellites are scaled to the peak intensities of the respective *hkl* reflections by the *hkl*-dependent intensity parameter *I*_sat_. The global satellite parameters, *m* ^sat^, σ^sat^, *s*_0_ and *I*_sat_, are optimized by the combined MC and ML optimization algorithms (Ribárik *et al.*, 2019 ▸).

**Figure 7:**
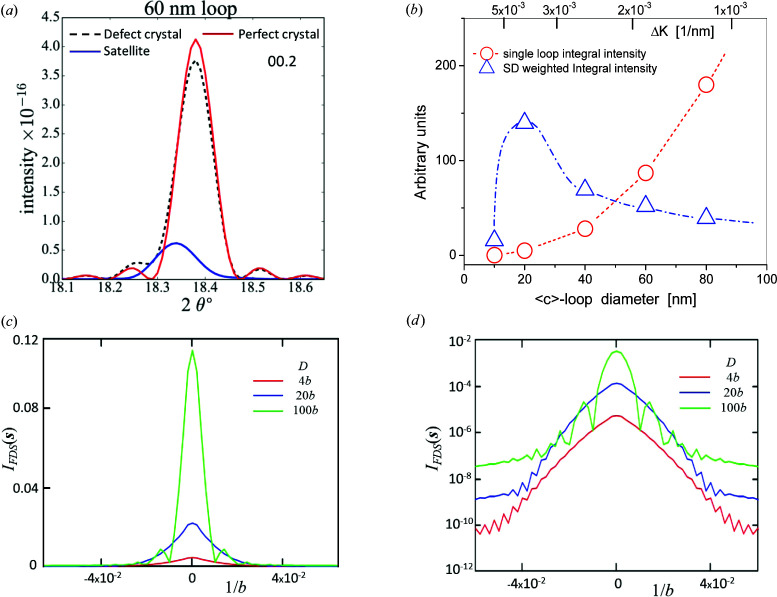
(*a*) Profiles of a 00.2 Zr reflection created in a 200 × 200 × 50 nm supercell by MD simulations. The red, dashed black and blue lines are the profiles of the perfect and the defect crystal and a satellite peak generated by the 60 nm 〈*c*〉-type vacancy loop, respectively. (*b*) Satellite integral intensities (open red circles) of 〈*c*〉-type vacancy loops and the size distribution (SD) weighted satellite integral intensities (open blue up-triangles) versus loop diameters, with power-law SD as suggested by Yi *et al.* (2015 ▸). (The dashed and dashed–dotted lines are only to guide the eye.) (*c*), (*d*) Calculated plots of the *I*_FDS_(**s**) peak profiles for **g** = (1,1,1) and dipole widths *D* = 4*b*, 20*b* and 100*b*. The *I*_FDS_(**s**) plots are shown (*c*) on a linear intensity scale and (*d*) on a logarithmic intensity scale.

On the basis of molecular dynamics (MD) simulations of 〈*c*〉-type loops, Hulse (2018 ▸) showed that (i) the integral intensity of single loops drops close to zero below a certain loop diameter and (ii) the offset of satellites from the main peak above a certain loop diameter decreases to the range of instrumental broadening when satellites blend into the main peak. Fig. 7 ▸(*a*) shows profiles of a 00.2 Zr reflection created in a 200 × 200 × 50 nm supercell by MD simulations (Hulse, 2018 ▸). The solid red and dashed black lines show the profiles of the perfect and the defect-containing crystal, respectively. The solid blue line shows the satellite peak generated by a single 60 nm diameter circular 〈*c*〉-type loop with the Burgers vector 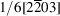. Fig. 7 ▸(*b*) shows satellite integral intensities (open red circles in arbitrary units) as a function of single 〈*c*〉-type loops on the basal plane versus loop diameters in pure Zr. Assuming a power-law size distribution of SDLs, as suggested by Yi *et al.* (2015 ▸), the size distribution weighted integral intensity is shown in Fig. 7 ▸(*b*) as blue up-triangles versus loop size. These data indicate that, below a certain loop size, the satellites merge into the long tail regions of the main peaks, whereas above a certain loop size they blend into the main peaks themselves. Although the presently available MD simulations of SDL satellites model 〈*c*〉 loops, these simulations reveal a qualitative relation between loop size and satellite visibility in diffraction patterns, indicating that satellites are affected only by a subset of SDLs. More elaborate MD simulations of loops generating satellites in powder diffraction patterns, including the effect of 〈*a*〉-type loops with prismatic habit planes, are in progress and will be published elsewhere. These MD simulations, together with the results shown in Fig. 5 ▸(*b*), indicate that there is a size window of loops that contribute to satellites. Loops smaller than the lower end of this window do not contribute to satellites, whereas loops larger than the higher end behave as ordinary lattice dis­locations, just broadening the main peaks.

### 2.5. Evaluation of partial dislocation densities related to 〈*a*〉- and 〈*c*〉-type dislocation loops and the true dislocation density   

The dislocation densities related to 〈*a*〉- and 〈*c*〉-type loops can be very different since they evolve at different stages of the irradiation process (Carpenter *et al.*, 1988 ▸; Harte *et al.*, 2017 ▸). On the basis of the difference in the contrast factors of the two different loop types, as calculated by Balogh *et al.* (2016 ▸) and shown in Fig. 3 ▸(*b*), the total dislocation density can be separated into partial values, ρ_〈*a*〉_ and ρ_〈*c*〉_, related to 〈*a*〉- and 〈*c*〉-type loops, respectively. The m.s.s., as indicated in equation (5), is proportional to 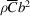. The true value of ρ and the effective value of *b* both depend on the fractions of the prevailing dislocation loop types. At first CMWP is run using fictional starting values for the Burgers vector b* and the scaling factor 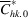. The partial m.s.s. values for the 〈*a*〉- and 〈*c*〉-type loops can be written as 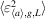 = 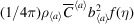 and 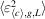 = 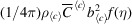, respectively, where we assume that *f*(η) is the same for all *hkl* peaks. The m.s.s. provided by CMWP, 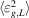 = 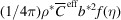, is considered as the measured value which is the sum of the two partial values corresponding to the 〈*a*〉- and 〈*c*〉-type loops:
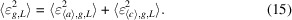
From this we obtain
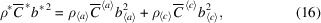
where 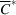 and ρ* are the contrast factor and the formal dislocation density given by the CMWP evaluation, respectively. The fractions φ_〈*a*〉_ and φ_〈*c*〉_ of the partial m.s.s. values are defined as
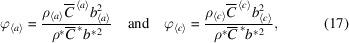
where φ_〈*a*〉_ + φ_〈*c*〉_ = 1. The partial dislocation densities are then
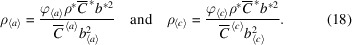



Equation (18) must hold for all *hkl*s and can be solved by the method of least squares for φ_〈*a*〉_ and φ_〈*c*〉_. The volume fractions of dislocation densities related to 〈*a*〉- and 〈*c*〉-type loops will be
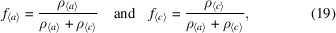
where ρ_〈*a*〉_ + ρ_〈*c*〉_ is the true dislocation density: ρ^true^ = ρ_〈*a*〉_ + ρ_〈*c*〉_. In the following, wherever dislocation densities are discussed or plotted we always mean the true values without using the superscript. The procedure in the above equations can be extended for lattice dislocations or lattice plus loop dislocations existing at the same time.

## 3. Results and discussion   

The proton irradiation dose in Zr and its alloys is calculated by the *SRIM* software package (Ziegler *et al.*, 2010 ▸). *SRIM* is a simulation method based on the energy deposited into the material and the damage is given in units of displacement per atom (dpa). Neutron irradiation dose is usually given in units of fluence, the number of neutrons per unit cross section penetrating the material: number of neutrons per unit area. In order to keep the genuine damage values, the two units will not be converted into each other unless necessary. When proton- and neutron-irradiated data are compared, the two units are matched by the conversion given by Griffiths (1988 ▸): 1 × 10^25^ n m^−2^ ≃ 1.67 dpa.

### 3.1. Fractions of 〈*a*〉- and 〈*c*〉-type dislocation loops   

**Figure 8:**
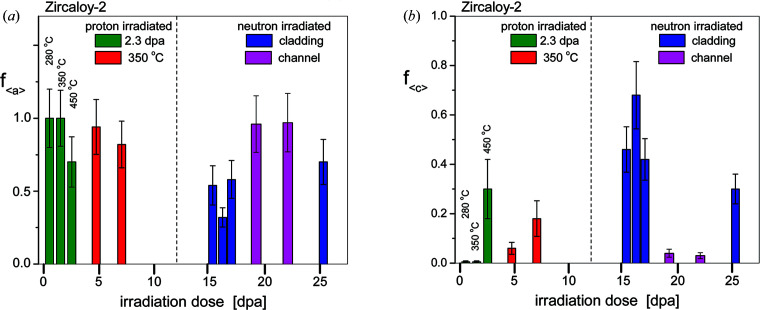
(*a*) Fractions of dislocation densities related to 〈*a*〉-type loops. (*b*) Fractions of dislocation densities related to 〈*c*〉-type loops. The green columns show data of the specimens proton-irradiated to 2.3 dpa at different temperatures. The red columns show data of the specimens proton-irradiated to different dose levels at 350°C. The blue and purple columns show data of the neutron-irradiated cladding and channel material specimens, respectively. Note the different scales in the two figures.

The fractions of 〈*a*〉- and 〈*c*〉-type dislocation loops are shown in Figs. 8 ▸(*a*) and 8 ▸(*b*), respectively. In the specimens proton-irradiated to 2.3 dpa at 280 or 350°C there are only 〈*a*〉-type loops. In the 450°C irradiated specimen the total dislocation density is very small (*f*_〈*a*〉_ < 1), indicating that at this higher temperature some 〈*c*〉 loops form even at relatively low dose levels. In the neutron-irradiated specimens of cladding material the small *f*_〈*a*〉_ values indicate a substantial number of 〈*c*〉 loops, in good correlation with the TEM observations of Harte *et al.* (2017 ▸). Fig. 6 of Harte *et al.* (2017 ▸) shows TEM micrographs of two of the same specimens investigated here (neutron-irradiated to 9.5 × and 14.7 × 10^25^ n m^−2^). The 〈*a*〉 and 〈*c*〉 loop fractions in Harte’s TEM micrographs are in good qualitative correlation with the present results.

### 3.2. Proton-irradiated Zircaloy-2 at different temperatures   

**Figure 9:**
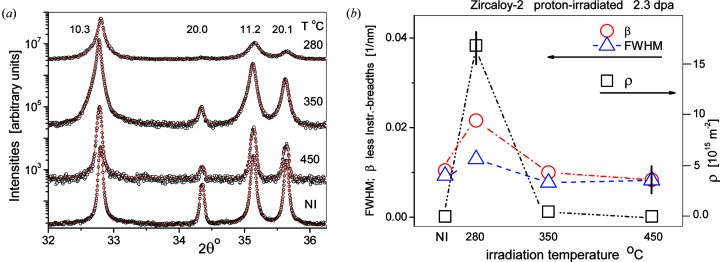
(*a*) Enlarged sections of measured (open circles) and CMWP-calculated (red lines) patterns, on a logarithmic intensity scale, of Zircaloy-2 specimens proton-irradiated to 2.3 dpa at 280, 350 and 450°C. For clarity the patterns are shifted vertically. NI stands for the ‘non-irradiated’ specimen. [In order to make the figure clearer, not all measured points (open circles) are shown in the plots.] (*b*) Open squares are the CMWP-provided dislocation densities ρ, and open circles and triangles are the instrumental corrected integral breadths β and FWHMs of the (10.3) peaks, plotted versus irradiation temperature. The lines are only to guide the eye. (The horizontal arrows refer to the different scales on the left and right.)

Enlarged sections of the measured and CMWP-calculated patterns of Zircaloy-2 specimens proton-irradiated to 2.3 dpa at 280, 350 and 450°C are shown in Fig. 9 ▸(*a*) with logarithmic intensity scales shifted relative to each other. The figure indicates that (i) the match between measured and CMWP-calculated patterns is good, and (ii) irradiation at 280°C causes the largest line broadening, which gradually becomes smaller with increasing irradiation temperature. The CMWP-evaluated dislocation densities ρ, the instrumental corrected integral breadth β and the FWHM values of the (10.3) peaks are shown versus the irradiation temperature in Fig. 9 ▸(*b*). There is only a weak correlation between the breadth values and the dislocation density. The breadth values change within a factor of about 2.8, whereas the dislocation densities change by a factor of about 320. Neither the FWHM nor the integral breadth β take into account the shape of the diffraction profiles correctly, which is so crucial in the Krivoglaz–Wilkens theory (Krivoglaz & Rjaboshapka, 1963 ▸; Wilkens, 1970 ▸) of line broadening from dislocated crystals. The analysis of breadths can be of valuable qualitative assistance in understanding the nature of line broadening, but it cannot provide quantitative results (Scardi *et al.*, 2004 ▸; Ribárik & Ungár, 2010 ▸).

**Figure 10:**
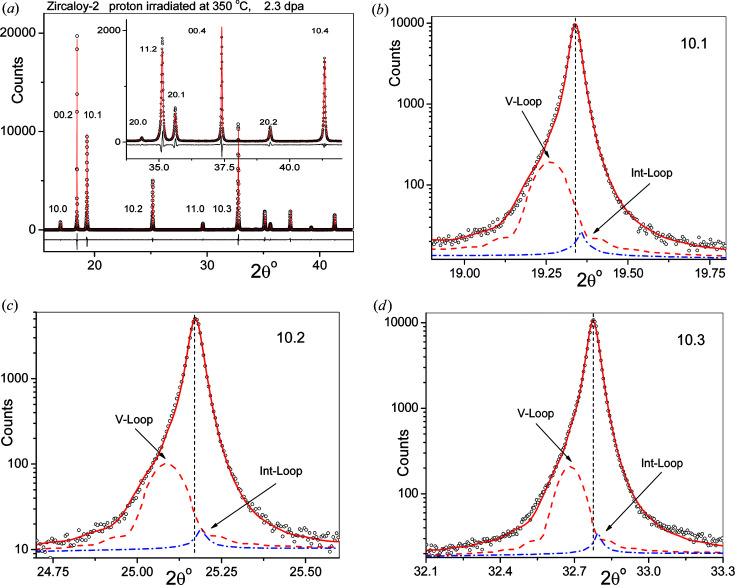
(*a*) Measured (open circles) and CMWP-calculated (red lines) diffraction patterns of proton-irradiated Zircaloy-2 at 350°C to a dose of 2.3 dpa. The inset is the enlarged higher-angle part of the pattern. The black lines at the bottom of the plots are the difference between the measured and calculated intensities. (*b*), (*c*), (*d*) Enlarged CMWP evaluations of the (10.1), (10.2) and (10.3) reflections, respectively. The satellite profiles are shown in the enlarged patterns. The red dashed and blue dashed–dotted lines are the satellite profiles of the vacancy- and interstitial-type 〈*a*〉 loops, respectively. (The diagonal arrows are for identifying the satellites.)

A typical CMWP evaluation of an X-ray diffraction pattern from proton-irradiated Zircaloy-2 specimens along with the satellites related to SDLs is shown in Fig. 10 ▸. The measured (open circles) and CMWP-calculated (red lines) patterns from the Zircaloy-2 specimen proton-irradiated at 350°C to 2.3 dpa are shown in Fig. 10 ▸(*a*). The inset is the enlarged higher-angle part of the pattern. Satellite peaks were evaluated according to the model described in Section 2.4. Figs. 10 ▸(*b*), 10 ▸(*c*) and 10 ▸(*d*) show enlarged profiles of the (10.1), (10.2) and (10.3) peaks on logarithmic intensity scales. The red dashed and blue dashed–dotted lines are the satellite profiles related to 〈*a*〉-type vacancy and interstitial character SDLs, respectively. The satellites related to interstitial loops are considerably smaller than those related to vacancy loops. This is most probably because, in Zr, interstitials migrate faster than vacancies (Christensen *et al.*, 2015 ▸), and therefore interstitial loops grow faster than vacancy loops and larger loops grow out more easily from the size window where satellites are generated.

### 3.3. Neutron-irradiated Zircaloy-2   

**Figure 11:**
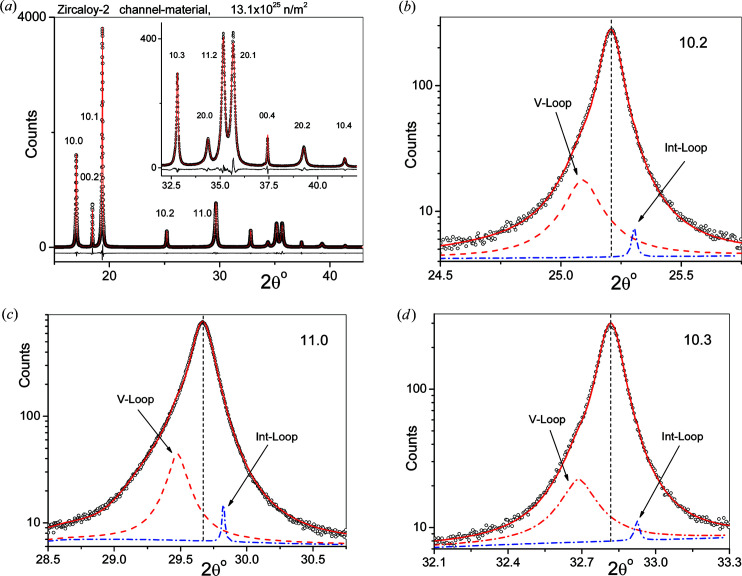
(*a*) Measured (open circles) and CMWP-calculated (red lines) diffraction patterns of neutron-irradiated Zircaloy-2 channel material, fluence = 13.1 × 10^25^ n m^−2^. The inset is the enlarged higher-angle part of the pattern. The black lines at the bottom of the plots are the difference between the measured and calculated intensities. (*b*), (*c*) and (*d*) Enlarged CMWP evaluations of the (10.2), (11.0) and (10.3) reflections, respectively. The satellite profiles are shown in the enlarged patterns. The red dashed and blue dashed–dotted lines are the satellite profiles of the vacancy- and interstitial-type 〈*a*〉 loops, respectively. (The diagonal arrows are for identifying the satellite profiles.)

A typical diffraction pattern of neutron-irradiated Zircaloy-2 taken from channel material is shown in Fig. 11 ▸(*a*). The inset is the enlarged higher-angle part of the pattern. The detailed CMWP fits along with the satellite patterns are shown for the (10.2), (11.0) and (10.3) reflections in Figs. 11 ▸(*b*), 11 ▸(*c*) and 11 ▸(*d*), respectively. The red dashed and blue dashed–dotted lines are the satellite profiles related to 〈*a*〉-type vacancy and interstitial character SDLs, respectively. The satellites for interstitial loops are considerably smaller than those of vacancy loops for the same reason as in the case of proton-irradiated specimens.

### 3.4. Dislocation densities and dipole character in proton- and neutron-irradiated specimens   

**Figure 12:**
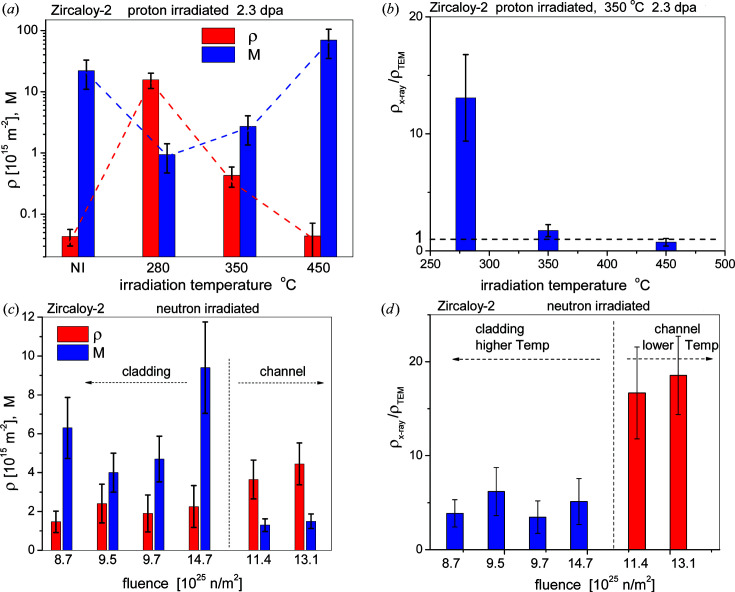
(*a*) Dislocation density (first bars, red) and *M* parameter values (second bars, blue) in a Zircaloy-2 alloy proton-irradiated to 2.3 dpa at 280, 350 and 450°C. The very first two bars on the far left of the figure correspond to the non-irradiated (NI) material. (*b*) Ratios of the CMWP- and TEM-determined dislocation densities, ρ_X-ray_/ρ_TEM_, of the same specimens as in (*a*) plotted versus temperature. TEM data were taken from Topping *et al.* (2019 ▸). (*c*) Dislocation densities (first bars, red) and *M* parameter values (second bars, blue) in neutron-irradiated Zircaloy-2 specimens plotted versus neutron fluence. The vertical dashed line separates the cladding and channel material data. (*d*) Ratios of the CMWP- and TEM-determined dislocation densities, ρ_X-ray_/ρ_TEM_, of the same specimens as in panel (*c*) plotted versus neutron fluence. TEM data were taken from Harte *et al.* (2017 ▸).

Dislocation densities and the *M* parameter values are shown for the Zircaloy-2 specimens proton-irradiated to 2.3 dpa at 280, 350 and 450°C in Fig. 12 ▸(*a*). In the non-irradiated state ρ is in the region of 10^13^ m^−2^ with *M* ≃ 22 ± 10, indicating an almost dislocation-free state. The large *M* value here means that the few dislocations are distributed randomly with no dipole character. Irradiation at the lowest temperature of 280°C produces a large dislocation density of about 1.64 × 10^16^ ± 0.2 × 10^16^ m^−2^ with a low value of *M* ≃ 0.9 ± 0.05. The small *M* value indicates a strong dipole character. At higher irradiation temperatures, the dislocation densities become smaller along with increasing *M* values, indicating weakening dipole character. The LPA results are in good qualitative correlation with the TEM micrographs in Fig. 2 ▸, showing a large density of small 〈*a*〉 loops at low irradiation temperatures, and substantially larger 〈*a*〉 loops with much lower density at higher irradiation temperatures. The ratio between X-ray- and TEM-determined dislocation densities, ρ_X-ray_/ρ_TEM_, for the proton-irradiated specimens is shown in Fig. 12 ▸(*b*), where the TEM data were taken from Topping *et al.* (2019 ▸). The dislocation densities and *M* parameter values in four cladding and two channel material Zircaloy-2 specimens neutron-irradiated to different fluences are shown in Fig. 12 ▸(*c*). The dislocation densities are smaller and the *M* values larger in the cladding materials than in the channel materials. Cladding structures usually operate at about 350°C, whereas channel structures operate at lower temperatures. According to Griffiths (1988 ▸), channel structures operate at temperatures between 300 and 320°C. Taking into account the operation temperature difference between cladding and channel materials, there is a striking similarity in the effect of irradiation temperature on proton- or neutron-irradiated materials. In both cases, ir­radiation at lower or higher temperature produces smaller or larger loops with larger or smaller ρ values and smaller or larger *M* values, respectively. The ratio between X-ray- and TEM-determined dislocation densities, ρ_X-ray_/ρ_TEM_, for the neutron-irradiated specimens is shown in Fig. 12 ▸(*d*), where the TEM data were taken from Harte *et al.* (2017 ▸).

Next, we discuss (i) the decrease in dislocation density and dipole character with temperature, and (ii) the difference between the dislocation densities obtained by X-ray LPA and TEM. The first issue can be understood by considering the competition between the nucleation and growth of dislocation loops. At lower temperatures the formation of new loops dominates over loop growth. Therefore, at lower temperatures, a larger number of smaller loops are present in the material. At higher temperatures loop growth dominates over loop formation, and probably Ostwald ripening also contributes to loop growth. Although the dislocation density at lower ir­radiation temperatures is larger, because of the smaller loop sizes the total number of point defects incorporated in loops is not necessarily larger in specimens irradiated at lower temperatures. The second issue is related to the different sampling and observation mechanisms in X-ray LPA and TEM investigations and to the possible loss of loops during thin-foil preparation. Enhanced prismatic loop mobility through free surfaces was discussed by Kroupa (1966 ▸) and shown via MD simulations by Mason *et al.* (2014 ▸) and Swinburne *et al.* (2016 ▸). Similar discrepancies between X-ray- and TEM-determined loop densities were found by Larson & Young (1987 ▸) and Larson (2009 ▸) in Cu-ion-irradiated Cu single crystals and by Sand *et al.* (2013 ▸) and Yi *et al.* (2015 ▸) in W^+^-ion-irradiated W. In these reports it was suggested that, while X-ray diffraction catches all loops irrespective of size, TEM observation is limited by the visibility criterion below a certain loop size. A more elaborate study resolving the apparent quantitative discrepancy between X-ray and TEM results is beyond the scope of the present work and has been submitted elsewhere (Ungár *et al.*, 2021 ▸).

### 3.5. Lattice constants of the matrix material in the presence of satellites   

Within first-order elasticity, dislocations do not change the lattice constants. However, in second-order elasticity, due to anharmonicity, they do cause a slight increase in lattice constants (Nabarro, 1967 ▸). Satellites produced by SDLs are small non-negligible diffraction peaks around major Bragg reflections. Different peak positions are shown in Fig. 6 ▸(*b*) in reciprocal-space coordinates, *K*, for the 10.2 peak of a Zircaloy-2 specimen proton-irradiated to 4.7 dpa at 350°C. The vertical dashed arrows from left to right indicate the peak positions related to vacancy-type satellites *K*_v-SDL_, the average peak position of the entire specimen *K*_av_, the peak position of the main peak determined by CMWP *K*_CMWP_ and the peak position related to interstitial-type satellites *K*_i-SDL_, respectively. The *K*_CMWP_, *K*_v-SDL_ and *K*_i-SDL_ values are given by the CMWP procedure for all measured reflections in the patterns. The lattice constants related to the main peaks, *a*_CMWP_ and *c*_CMWP_, can be obtained using the Hull–Davey equation (Hull & Davey, 1921 ▸) using the *K*_CMWP_ peak positions. The changes in the average lattice constants *a*_av_ and *c*_av_ relative to the main-peak values *a*_CMWP_ and *c*_CMWP_ can be obtained from the satellite shifts 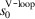 and 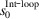 related to the pure prismatic (11.0) and pure basal (00.2) reflections using equations (14*a*)[Disp-formula fd14a] and (14*b*)[Disp-formula fd14b]:
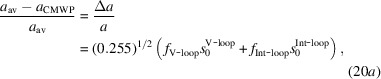


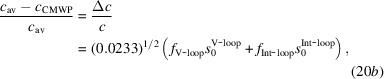
where *f*_V-loop_ and *f*_Int-loop_ are the integral intensities of satellites relative to the main peaks, and 0.255 and 0.0233 are the contrast factors of dislocation loops in the (11.0) and (00.2) directions, respectively, as calculated by Balogh *et al.* (2016 ▸) and shown in Fig. 2 ▸(*c*). The change in unit-cell volume is
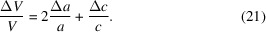



**Figure 13:**
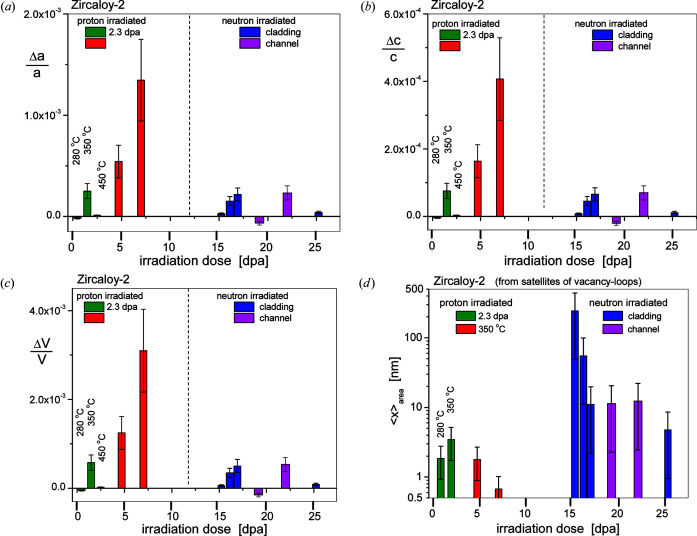
The effect of satellites on cell parameters. Relative changes in the (*a*) *a* and (*b*) *c* parameters. (*c*) The relative change in unit-cell volumes. The blue and purple columns show data of the neutron-irradiated cladding and channel material specimens. (Note that both Δ*a*/*a* and Δ*c*/*c* are expansions, except in the case of the lower-dose neutron-irradiated channel material specimen and the specimen proton-irradiated at 280°C to 2.3 dpa.) (*d*) The area average mean size 〈*x*〉_area_ of vacancy-type loops obtained from the median *m* and variance σ of the log-normal size distribution function in the satellite profile function for the proton- and neutron-irradiated Zircaloy-2 specimens. The green columns show data of the specimens proton-irradiated to 2.3 dpa at different temperatures. The red columns show data of the specimens proton-irradiated to different dose levels at 350°C. The blue and purple columns show data of the neutron-irradiated cladding and channel material specimens.

The changes in lattice constants and unit-cell volume, Δ*a*/*a*, Δ*c*/*c* and Δ*V*/*V*, caused by the presence of satellites are shown in Fig. 13 ▸. The related peak position *K*_av_ is the second vertical arrow from the left in Fig. 6 ▸(*b*). Rietveld procedures give these values by definition. The average (*a*_av_ and *c*_av_) and main-peak (*a*_CMWP_ and *c*_CMWP_) lattice constants are related as
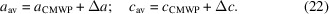



Satellites can shift the average lattice constants due to the strained volumes around SDLs. Therefore, solute element concentrations can be better determined from the *a*_CMWP_ and *c*_CMWP_ values. Fig. 13 ▸ shows that the lattice-parameter and unit-cell volumes vary over relatively wide ranges, between about 1 × 10^−5^ and 3 × 10^−3^ for the proton- and between about −5 × 10^−5^ and 5 × 10^−4^ for the neutron-irradiated specimens. The area average mean sizes 〈*x*〉_area_ of vacancy-type loops obtained from the median and variance, *m*_sat_ and σ_sat_, of the log-normal size distribution function in the satellite profile function are shown in Fig. 13 ▸(*d*). The proton-irradiated values vary between about 0.7 ± 0.3 and 3.5 ± 1.7 nm, whereas the neutron-irradiated values vary between about 5 and 250 nm. The two largest 〈*x*〉_area_ values are 244 ± 200 and 55 ± 40 for the data at 14.5 and 15.4 dpa (8.7 and 9.5 × 10^25^ n m^−2^ fluence, respectively). Although the median values for these two samples are small, *i.e.* *m*_sat_ = 0.27 ± 0.15 and 2.14 ± 1.2 nm, the variance values are large: σ_sat_ = 1.65 ± 0.5 and 1.14 ± 0.3, respectively. According to equation (3), the large σ_sat_ values give large exponential factors of 903 ± 300 and 26 ± 8, giving large 〈*x*〉_area_ values for these two samples. 〈*c*〉 loops are of vacancy type; therefore vacancy-type 〈*a*〉 and 〈*c*〉 loops generate overlapping satellites. Since 〈*c*〉 loops are substantially larger than 〈*a*〉 loops (Harte *et al.*, 2017 ▸), it is not possible to interpret the size of 〈*a*〉 loops from satellites when the two loop types are present at the same time. A further issue of getting the total size distribution from satellites is, as shown in Fig. 7 ▸(*b*), that satellites are affected only by a subset of SDLs. Determination of the size distribution of irradiation-induced dislocation loops is beyond the scope of the present work, but we are working on the problem and have submitted this elsewhere (Ungár *et al.*, 2021 ▸).

## 4. Conclusions   

We have developed a systematic X-ray line profile analysis procedure to determine the total dislocation density and dipole character related to irradiation-induced dislocation loops in neutron- or proton-irradiated commercial polycrystalline Zr alloys. X-ray LPA has long been developed as a powerful tool to determine the dislocation densities and character in plastically deformed crystalline materials, in good agreement with TEM analysis. In the CMWP procedure the strain part of the line broadening is evaluated using the two-parameter strain function, *f*(η), developed by Wilkens, providing the total dislocation density ρ and the dislocation arrangement parameter *M*. However, neutron- or proton-irradiated commercial polycrystalline Zr alloys reveal challenging issues. (i) Peak broadening is more pronounced in the tail regions of peaks than in the FWHM. (ii) Small satellites can appear around the main Bragg peaks. (iii) The dislocation densities can reach a level of 10^17^ m^−2^, along with *M* parameter values well below unity. (iv) The total dislocation density given by X-ray LPA is usually larger than the value obtained from TEM micrographs.

Our results show that, in powder diffraction patterns of irradiated materials, while the FWHMs change by less than a factor of five, the dislocation density, in which the *M* parameter plays a decisive role, can change by a factor of several hundred. The long tail regions of the peaks of irradiated materials correlate with the strong dipole character of SDLs.

On the basis of theoretical considerations, we have shown that the total dislocation density can be obtained from the main diffraction peaks free from small satellites. We extended the CMWP procedure for fitting satellite peaks around Bragg reflections in order to exclude them from the main peaks in the procedure of determining the total dislocation densities. We justify this procedure by referring to the theoretical description of asymmetric peak profiles generated by heterogeneous microstructures, where it was shown that the main symmetric part of the profiles provides the total dislocation density. Referring to the wide size distribution of SDLs, we model the satellites by size-broadened peak profiles assuming a log-normal size distribution.

In previous work, Seymour *et al.* (2017 ▸) reported that TEM-determined dislocation densities in neutron-irradiated channel materials were smaller, and X-ray values larger, than in cladding materials. We have explained this apparent discrepancy by taking into account that (i) at lower irradiation temperatures smaller loops form with larger densities and (ii) X-ray LPA gives the total dislocation density including that related to the smallest loops, whereas in TEM micrographs it can become difficult to observe and count dislocation loops below a certain loop size, especially when the loop density is large.

On the basis of average contrast factors, we developed a procedure to determine the fraction of 〈*a*〉- and 〈*c*〉-type dislocation loops. We have found that higher irradiation temperatures favor 〈*c*〉-loop formation. Our results show that LPA of powder diffraction patterns is a valuable tool, complementing TEM, to determine different qualitative and quantitative properties of the microstructure in irradiated materials.

## 5. Related literature   

For further literature related to the supporting information, see Aitchinson & Brown (1957 ▸), Arley & Buch (1950 ▸), Balogh *et al.* (2006 ▸, 2009 ▸, 2010 ▸), Bertaut (1949 ▸), Cordier *et al.* (2004 ▸), Dragomir & Ungár (2002*b* ▸), Gaál (1973 ▸, 1976 ▸, 1984 ▸), Gillies & Lewis (1968 ▸), Guinier (1963 ▸), Hall (1949 ▸), Jakobsen *et al.* (2006 ▸, 2007 ▸), Kocks & Scattergood (1969 ▸), Krill & Birringer (1998 ▸), Langford & Wilson (1978 ▸), Langford *et al.* (1993 ▸, 2000 ▸), Martinez-Garcia *et al.* (2008 ▸), Ódor *et al.* (2020 ▸), Ribárik *et al.* (2001 ▸, 2004 ▸), Scherrer (1918 ▸), Stokes & Wilson (1944 ▸), Terwilliger & Chiang (1995 ▸), Ungár *et al.* (2005 ▸, 2010 ▸), Valiev *et al.* (1994 ▸), Wilkens (1969 ▸), Williamson & Hall (1953 ▸) and York (1999 ▸).

## Supplementary Material

Mathematical principles. DOI: 10.1107/S1600576721002673/nb5274sup1.pdf

Manual for CMWP. DOI: 10.1107/S1600576721002673/nb5274sup2.pdf

Manual for CMWP Part 2. DOI: 10.1107/S1600576721002673/nb5274sup3.pdf

Additional figures. DOI: 10.1107/S1600576721002673/nb5274sup4.pdf
