# Supplementary material for: Characterizing dislocation loops in irradiated polycrystalline Zr alloys by X-ray line profile analysis of powder diffraction patterns with satellites
Source: J Appl Crystallogr. 2021 May 25;54(Pt 3):803–21. doi: 10.1107/S1600576721002673 (PMC8202032; doi:10.1107/S1600576721002673)
Supplement: Supplementary file 1 [file j-54-00803-sup1.pdf]

Supplement S1

Principles for a working-map of the Convolutional-Multiple-Whole-Profile (CMWP) X-ray and neutron line profile analysis procedure

Gábor Ribárik and Tamás Ungár

1. Line profile parameters

The X-ray diffraction measurements usually provide the $I(2\theta)$ intensity profiles of the different reflections. In order to compare this with theory, it is practical to convert the variable 2θ to g, the variable of the reciprocal space, where $g=2\sin\theta/\lambda$ is the absolute value of the diffraction vector. The value of g at $2\theta_B$, the exact Bragg position, is denoted by g_B and it is expressed as: $g_B=2\sin\theta_B/\lambda$. In the following often g (a general position in reciprocal space) will be denoted by K. The variable of the intensity profiles can be expressed as:

$$s = g - g_B \approx (2\cos\theta_B/\lambda)\Delta\theta \ . \qquad (1)$$

The most important characteristic parameters of an $I(s)$ intensity function corresponding to the Bragg peak at $2\theta_B$ are:
(1) the maximum intensity:

$$I_0 = \max\{I(s) \mid s \in \mathbb{R}\}, \qquad (2)$$

(2) the Full Width at Half Maximum (FWHM):

$$\mathrm{FWHM}\{I(s)\} = s_2 - s_1, \text{ where } s_1 < s_2 \text{ and } I(s_1) = I(s_2) = I_0/2 \ , \qquad (3)$$

(3) the integral breadth (equivalent to the area of the normalized intensity curve):

$$\beta = \frac{1}{I_0}\int_{-\infty}^{\infty} I(s)ds \ . \qquad (4)$$

In X-ray or neutron diffraction usually FWHM < β.

2. Size broadening of diffraction profiles

With decreasing scattering volume the diffraction profiles broaden. This is called size broadening. X-ray or neutron diffraction profiles provide the coherently scattering domain size or crystallite size (Bertaut, 1950). This is often smaller than the grain size obtained by transmission electron microscopy (TEM). In Ungár et al. (S2005) it was shown that the coherently scattering domain size given by X-ray or neutron line profile analysis provides the subgrain or dislocation cell size bounded by small angle grain boundaries or dipolar walls. In the present section the effect of the coherently scattering domain size on the diffraction profiles is discussed.

2.1 Size parameters

The definition of the commonly used different size parameters, given in eqs. (12), (13) and (16) below, is presented through a simple case study (Warren, 1969). Let's assume an infinite crystallite plane with the thickness of N atoms. According to the theory of kinematical X-ray scattering, the line profile of this special crystallite is:

$$I(s) \sim \frac{sin^2(\mathrm{N}x)}{sin^2(x)}\ , \qquad (5)$$

where $x = \pi\mathbf{Ga}$, $\mathbf{G} = \mathbf{g}+\Delta\mathbf{g}$, $\mathbf{g}$ is the diffraction vector, $\Delta\mathbf{g}$ is a small vector, and **a** is the unit cell vector chosen to be perpendicular to the plane of the crystallite. The function $\sin^2(\mathrm{Nx})/\sin^2(\mathrm{x})$ describes the shape and position of the peaks in this special case. This function is plotted for different values of N in Fig. 1. It has a maximum value at positions: $x = n\pi$, $n \in \mathbb{Z}$. This condition

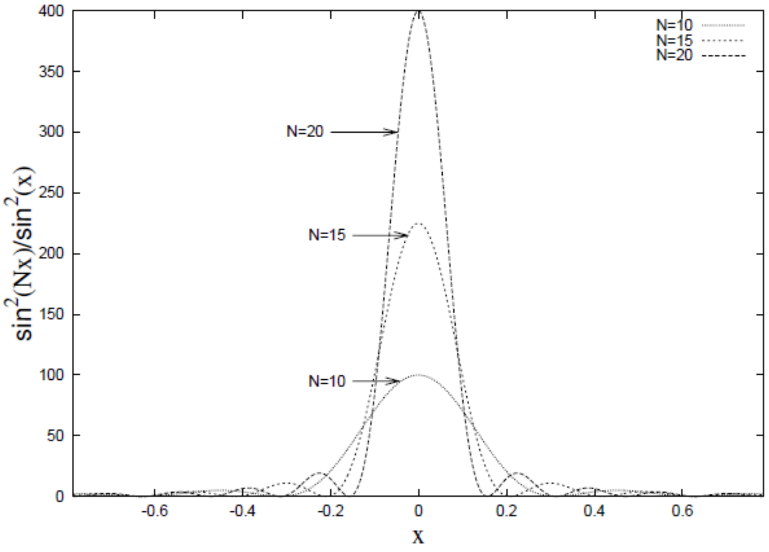

Fig. 1. The function $\sin^2(\mathrm{Nx})/\sin^2(\mathrm{x})$ plotted close to its first maximum for different values of N. As N tends to infinity, the curve becomes a delta function.

is equivalent to the Laue equations. The maximum value of this function is $\lim_{x\to 0} \frac{sin^2 Nx}{sin^2 x} = \mathrm{N}^2$. For large values of N this profile function can be approximated by the following simple function:

$$\frac{sin^2 Nx}{sin^2 x} = \mathrm{N}^2\left(\frac{sinNx}{Nx}\right)^2\ . \qquad (6)$$

The FWHM value of this function is given by:

$$\frac{sinNx}{Nx} = \frac{1}{\sqrt{2}}\ . \qquad (7)$$

This transcendent equation can be numerically solved for Nx: the solution is $Nx = 1.39$. Consequently, the FWHM, i.e. $2x$, is reciprocally proportional to the number of lattice points perpendicular to the diffracting plane:

$$\mathrm{FWHM} = 2.78\frac{1}{N}\,. \qquad (8)$$

This means that the profile function becomes narrower as the crystallite becomes thicker. The integral breadth of this curve is:

$$\beta = \frac{1}{N^2}\int_{-\infty}^{\infty}\frac{\sin^2 Nx}{x^2}\,dx = \pi\frac{1}{N}\,. \qquad (9)$$

A conventional θ - 2θ diffractometer measures the intensity parallel to the direction of the **g** diffraction vector as a function of $s = |\Delta\mathbf{g}|$. The FWHM and integral breadth values of the $I(s)$ intensity function as a function of s can be expressed as:

$$\mathrm{FWHM} = \frac{2.78}{\pi}\frac{1}{Na\cos((\mathbf{G},\mathbf{a})\measuredangle)} = \frac{0.9}{L_{\mathbf{G},\mathbf{a}}}\,, \qquad (10)$$

$$\beta = \frac{1}{Na\cos((\mathbf{G},\mathbf{a})\measuredangle)} = \frac{1}{L_{\mathbf{G},\mathbf{a}}}\,, \qquad (11)$$

where $L_{\mathbf{G},\mathbf{a}} = Na\cos((\mathbf{G},\mathbf{a})\measuredangle)$ is the projection of the crystallite width in the direction of the diffraction vector. This means that by measuring the widths of the peak profiles, only the crystallite size parallel to the diffraction vector can be determined. For the reflection *h00*, $L_{\mathbf{G},\mathbf{a}}$ is equal to the thickness of the crystal, i.e. either to 0.9/FWHM or to 1/β, respectively. The following two size parameters can be defined generally for an arbitrary $I(s)$ intensity profile:

$$D = \frac{0.9}{\mathrm{FWHM}}\,, \qquad (12)$$

or

$$d = \frac{1}{\beta}\,. \qquad (13)$$

Eq. (12) is the Scherrer (S1918) equation. The more rigorous theoretical description is done by the Fourier transform of the intensity profile Warren (1959):

$$\begin{cases} \pi(N-\pi|L|), & \text{if } |L| \le \dfrac{N}{\pi} \\ 0, & \text{if } |L| > \dfrac{N}{\pi}. \end{cases} \qquad (14)$$

Fig. 2 shows this function.

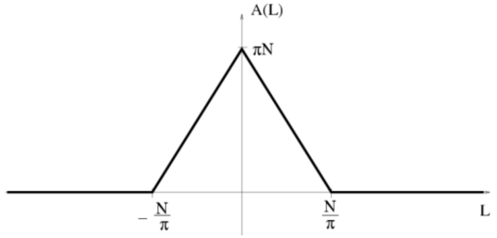

Figure 2: The Fourier transform of the $\sin^2(Nx)/x^2$ function. For an infinite-plane crystallite of the thickness of N atoms the Fourier transform of the $I(s)$ size function is:

$$\begin{cases} \frac{N}{L_{\mathbf{G},\mathbf{a}}}\left(N - \frac{N}{L_{\mathbf{G},\mathbf{a}}}|L|\right), & \text{if } |L| \le L_{\mathbf{G},\mathbf{a}} \\ 0, & \text{if } |L| > L_{\mathbf{G},\mathbf{a}}. \end{cases} \quad (15)$$

Eq. (15) shows that that $L_{\mathbf{G},\mathbf{a}}$ can be determined from the initial slope of the Fourier transform. The size parameter L_0 is generally defined for an arbitrary $I(s)$ intensity profile as the initial slope of the $A^S(L)$ Fourier transform (Warren, 1959):

$$-\frac{A_S(0)}{L_0} = \frac{\mathrm{d}}{\mathrm{d}L} A_S(L)\bigg|_{L=0}. \quad (16)$$

The definition of L_0 is illustrated in Fig. 6 below for the case of spherical crystallites with lognormal size distribution. The different size parameters usually satisfy the following relation (Langford & Wilson, S1978): $D \ge d \ge L0$. For spherical crystallites d and L_0 are proportional to the volume- and area-weighted average crystallite sizes, respectively (Langford & Wilson, S1978):

$$< x >_{vol} = \frac{\sum_i V_i d_i}{\sum_i V_i} = \frac{4}{3} d \quad , \quad (17)$$

$$< x >_{area} = \frac{\sum_i A_i d_i}{\sum_i A_i} = \frac{3}{2} L_0 \quad . \quad (18)$$

2.2. Size distribution functions

In the previous section the size broadening of a single crystallite has been discussed. A polycrystalline or fine powder sample consists of many crystallites with different sizes where the size distribution function has to be implemented. By selecting the proper size distribution and assuming a realistic crystallite shape, the size broadened profile can be calculated on a

theoretical basis. Several distribution functions can be used to describe the size distribution of crystallites (Langford et al., S2000; Scardi & Leoni, S2002). Among these, one of the most flexible one is the lognormal size distribution (Aitchison & Brown, S1957). The validity of this function was confirmed by several observations and successful applications (Valiev et al., S1994; Terwilliger & Chiang, S1995; Krill & Birringer, S1998, Ungár et al., S1999; Langford et al., S2000). The Gamma distribution (Arley & Buch, S1950) is also suitable to describe the experimental size distributions. York (S1999) proposed another distribution. These distributions are discussed below.

(i) *The lognormal distribution*. According to TEM observations (Aitchison & Brown, S1957) this is the most commonly used size distribution of particle size in a fine powder. It can be shown that a milling procedure leads to a lognormal size distribution (Hinds, S1982). Therefore, the lognormal distribution is widely used in microstructural investigations. It is obtained by substituting the variable of a normal distribution with its logarithm. When one applies the lognormal distribution to describe the size distribution of crystallites, this means that the logarithm of the crystallite size follows a normal distribution. The density function of the lognormal size distribution has the following form:

$$f(x) = \frac{1}{\sqrt{2\pi}\sigma}\frac{1}{x}\exp\left[-\frac{\left(\log\left(\frac{x}{m}\right)\right)^2}{2\sigma^2}\right]. \qquad (19)$$

where m and σ are the parameters of the distribution, log$m$ is the median and σ the variance of the *normal* distribution. The parameters m and σ are called “median” and “variance” of the *lognormal* size distribution. In the CMWP procedures (Ribárik et al. S2001; Ribárik et al. S2004; Ungár et al. S2001) b=log$m$ and c=σ√2 are the parameters used for the optimization procedure.

(ii) *The Gamma distribution*. The Gamma distribution (Arley & Buch, S1950) is also flexible and can be used for describing crystallite size distributions. Its density function has the form:

$$f(x) = \frac{a}{b\Gamma(a)}\left(\frac{ax}{b}\right)^{a-1}\exp\left(-\frac{ax}{b}\right), \qquad (20)$$

where a and b are the parameters of the distribution and Γ(x) is the Gamma function.

(iii) *York’s distribution*. The York distribution (York, S1999) was obtained by assuming a normal growth phenomena. The density function of the York distribution has a form similar to the Gamma distribution:

$$f(x) = \frac{1}{b\Gamma(a)}\left(\frac{ax}{b}\right)^{a}\exp\left(-\frac{ax}{b}\right), \qquad (21)$$

where a and b are the parameters of the distribution and Γ(x) is the Gamma function.

We note that Leoni & Scardi (S2004) proposed a bar-diagram for the size distribution density function and each individual column height of this diagram is fitted independently in their pattern-refining procedure. Since this is an ad-hoc, experimental distribution its discussion is beyond the scope of this theoretical section. Langford et al. (S2000) have shown that the above discussed size distribution functions correlate well with experimental X-ray diffraction profiles and, that it is difficult, if not impossible, to distinguish between size distribution functions on an experimental basis.

2.3. Determination of the size profile (Ribárik et al. S2001; Ribárik et al. S2004; Ungár et al. S2001)

Assuming a particular crystallite shape and crystallite size distribution, one can determine the theoretical size profile. Bertaut (S1949) and Guinier (S1963) have shown, that the size profile of a powder specimen consisting of crystallites with arbitrary size and shape can be determined as follows:

(a) the crystallites should be divided into columns parallel to the diffraction vector **g**,
(b) the “size profile” is obtained as the volume-weighted sum of the intensity profiles normalized by their integral intensities corresponding to each column. The size profile of a column with area A_i and height M_i, normalized by its integral intensity:

$$\frac{\sin^2(M_i \pi s)}{M_i (\pi s)^2} . \qquad (22)$$

By summing up the contributions from all columns of all crystallites using the volume of the column as weight, the intensity distribution becomes:

$$I(s) \sim \sum_i \frac{\sin^2(M_i \pi s)}{M_i (\pi s)^2} A_i M_i . \qquad (23)$$

Introducing g(M)dM as the sum of the volumes of the columns with height between M and M+dM of all crystallites:

$$g(M)\,\mathrm{d}M = \sum_j \mathrm{d}V_j(M, \mathrm{d}M) . \qquad (24)$$

Using this quantity, the intensity distribution can be written as:

$$I(s) \sim \int_0^\infty \frac{\sin^2(M \pi s)}{M (\pi s)^2} g(M)\,\mathrm{d}M , \qquad (25)$$

Eq. (25) shows that the size profile can be obtained by determining $g(M)\mathrm{d}M$, which depends on the crystallite shape and the size distribution of the crystallites. Now we calculate the size profile according to the lognormal size distribution for (i) spherical and (ii) ellipsoidal crystallite shapes (Ribárik et al. S2001; Ungár et al. S2001). Latter is a simple and general description of deviations from spherical shape. It also accounts for anisotropic size broadening as a function of *hkl*. For a particular crystallite shape $g(M)\mathrm{d}M$ is determined first for one crystallite. The calculation is based on the geometrical properties of the crystallite shape. The size profile is obtained by summing up for all crystallites using the crystallite size distribution function. In the case of *spherical* crystallites and *lognormal* size distribution, $g(M)\mathrm{d}M$ is obtained as:

$$x^2 = y^2 + \left(\frac{M}{2}\right)^2 , \qquad (26)$$

where we used the notations of Fig. 3.

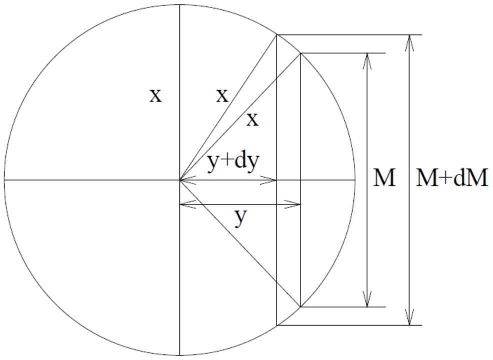

Figure 3. Determination of g(M)dM in the case of a spherical crystallite with radius x. The goal is to calculate the volume of the part of the sphere with column length between M and M+dM. This part of the sphere is approximated by an annulus based prism and it is expressed with M, x and y, where y is the radius of the annulus. For one crystallite $g(M)\mathrm{d}M$ is equal to the volume of the part of the sphere with column length between M and $M+dM$:

$$g(M)\mathrm{d}M \approx -2py\mathrm{d}yM. \qquad (27)$$

Differentiating eq. (26) we obtain: $2y\mathrm{d}y = -M\mathrm{d}M/2$. Therefore for *one* crystallite:

$$g(M)\mathrm{d}M \sim M2\ \mathrm{d}M. \qquad (28)$$

Since $f(x)\mathrm{d}x$ is proportional to the number of the crystallites with diameter between x and $x+\mathrm{d}x$ and all the crystallites with diameter $x \geq M$ contain the column length M:

$$g(M)\,\mathrm{d}M \sim \left(\int_M^{\infty} f(x)\,\mathrm{d}x\right) M^2\,\mathrm{d}M . \qquad (29)$$

Using the distribution density function in eq. (19), this integral can be expressed as:

$$\int_{M}^{\infty} f(x)\,\mathrm{d}x = \frac{1}{2}\,\mathrm{erfc}\left[\frac{\log\left(\frac{M}{m}\right)}{\sqrt{2}\sigma}\right], \qquad (30)$$

where erfc is the complementary error function:

$$\mathrm{erfc}(x) = \frac{2}{\sqrt{\pi}}\int_{x}^{\infty} e^{-t^2}\,\mathrm{d}t . \qquad (31)$$

For *all* crystallites in the specimen $g(M)\mathrm{d}M$ can be written as:

$$g(M)\,\mathrm{d}M \sim M^2\,\mathrm{erfc}\left[\frac{\log\left(\frac{M}{m}\right)}{\sqrt{2}\sigma}\right]\mathrm{d}M . \qquad (32)$$

Using eq. (25) the size profile is obtained (Ribárik et al., S2001):

$$I^S(s) = \int_{0}^{\infty} M\,\frac{\sin^2(M\pi s)}{(\pi s)^2}\,\mathrm{erfc}\left[\frac{\log\left(\frac{M}{m}\right)}{\sqrt{2}\sigma}\right]\mathrm{d}M . \qquad (33)$$

This size profile-function is plotted for different values of m and σ in Figs. 4 and 5.

The CMWP procedure is working in Fourier space (Ribárik et al. S2001; Ribárik et al. S2004). Now we determine the Fourier transform of the size profile. According to eq. (14), the Fourier transform of the $\sin^2(M\pi s)/(\pi s)$ function is:

$$\begin{cases} M-|L|, & \text{if } |L|\le M \\ 0, & \text{if } |L|>M \end{cases} . \qquad (34)$$

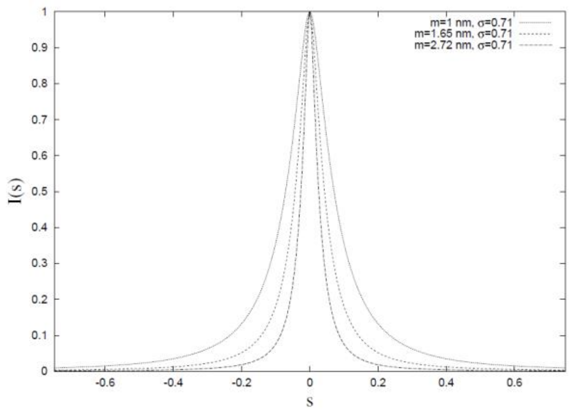

Figure 4: The size function for spherical crystallites with lognormal distribution with fixed value of σ = 0.71, as a function of s. The value of *m* varies for the different curves. The value of *m* is indicated in the upper right corner of the figure.

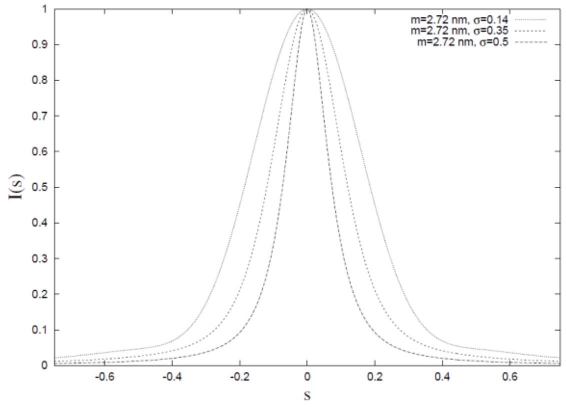

Figure 2.5: The size function for spherical crystallites with lognormal distribution with fixed value of *m* = 2.72 nm, as a function of *σ*. The value of σ varies for the different curves. The value of σ is indicated in the upper right corner of the figure.

Using eq. (34) the Fourier transform of the size profile-function in eq. (33) can be given as:

$$
\begin{aligned}
A^S(L) &= 2\int_0^\infty I^S(s)\cos(2\pi sL)\,\mathrm{d}s = \\
&= 2\int_0^\infty \left(\int_0^\infty M\,\frac{\sin^2(M\pi s)}{(\pi s)^2}\,\mathrm{erfc}\left[\frac{\log\left(\frac{M}{m}\right)}{\sqrt{2}\sigma}\right]\mathrm{d}M \right)\cos(2\pi sL)\,\mathrm{d}s = \\
&= \int_0^\infty M\left(2\int_0^\infty \frac{\sin^2(M\pi s)}{(\pi s)^2}\cos(2\pi sL)\,\mathrm{d}s\right)\mathrm{erfc}\left[\frac{\log\left(\frac{M}{m}\right)}{\sqrt{2}\sigma}\right]\mathrm{d}M = \\
&= \int_{|L|}^\infty \left(M^2 - |L|M\right)\mathrm{erfc}\left[\frac{\log\left(\frac{M}{m}\right)}{\sqrt{2}\sigma}\right]\mathrm{d}M.
\end{aligned}
\qquad (35)
$$

Using substitutions and partial integration the above integral can be simplified:

$$A^S(L) = \frac{m^3 \exp(4.5\sigma^2)}{3} erfc\left[\frac{\ln(|L|/m)}{\sqrt{2}\,\sigma} - 1.5\sqrt{2}\sigma\right] -$$
$$- \frac{m^2 \exp(2\sigma^2)|L|}{2} erfc\left[\frac{\ln(|L|/m)}{\sqrt{2}\,\sigma} - \sqrt{2}\sigma\right] + \frac{|L|^3}{6} erfc\left[\frac{\ln(|L|/m)}{\sqrt{2}\,\sigma}\right]. \qquad (36)$$

Dividing $A^S(L)$ by its maximum value, the normalized size Fourier transform is obtained. The maximum value of $A^S(L)$ is:

$$A^S(0) = \frac{2m^3 \exp\left(\frac{9}{4}(\sqrt{2}\sigma)^2\right)}{3} \qquad (37)$$

An example for the plot of the size Fourier transform is shown in Fig. 2.6.

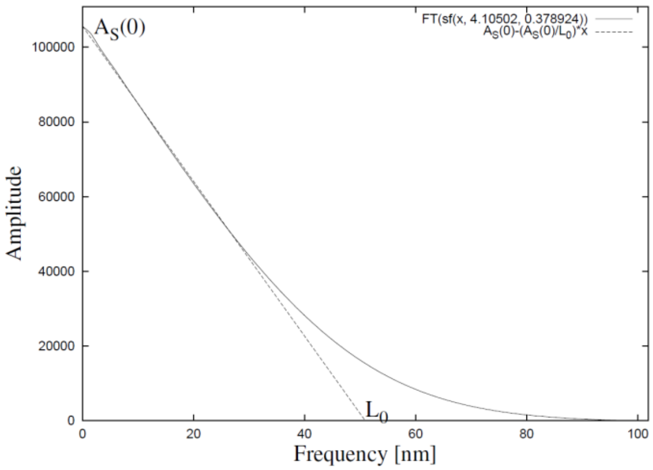

Figure 6. The theoretical Fourier transform of a size profile for spherical crystallites with lognormal size distribution. The initial slope defining L_0 is also indicated in the figure.

We note that the Fourier transform of the size profile can also be obtained from the Patterson function of the scattering object (Guinier, S1963; Zilahi et al., S2016):

$$A^S(\mathbf{L}) = \frac{1}{V}\int \sigma(\mathbf{r})\sigma(\mathbf{r}+\mathbf{L})\,\mathrm{d}^3\mathbf{r} , \qquad (38)$$

where $\sigma(\mathbf{r})=1$ if $\mathbf{r} \in$ *“scattering object”* otherwise $\sigma(\mathbf{r})=0$. The two different calculations provide

exactly the same result for $A^S(L)$ (Ribárik et al., S2001). The different size parameters can be obtained as functions of m and σ using the explicit forms of $I^S(s)$ and $A^S(L)$. The derivation of $A^S(L)$ gives the L_0 size parameter:

$$L_0 = -\frac{A^S(0)}{\left(\frac{\mathrm{d}A^S(L)}{\mathrm{d}L}\right)_{L=0}} = \frac{2m\exp\left(\frac{5}{4}(\sqrt{2}\sigma)^2\right)}{3} \quad . \tag{39}$$

The maximum value of the size profile is:

$$I^S(0) = \int_0^\infty M^3 \operatorname{erfc}\left[\frac{\log\left(\frac{M}{m}\right)}{\sqrt{2}\sigma}\right] \mathrm{d}M \quad . \tag{40}$$

The integral of the normalized size profile gives the size parameter d:

$$d = \frac{1}{\beta} = \frac{I^S(0)}{\int_{-\infty}^{\infty} I^S(s)\,\mathrm{d}s} = \frac{I^S(0)}{A^S(0)} = \frac{3m\exp\left(\frac{7}{4}(\sqrt{2}\sigma)^2\right)}{4} \quad . \tag{41}$$

The arithmetic-, area- and volume-weighted mean crystallite size values are:

$$\langle x\rangle_j = m\exp(k\sigma^2), \tag{42}$$

2.4. Anisotropic size broadening (Ribárik et al. S2001; Ribárik et al. S2004; Ungár et al. S2001)

If the crystallite shape is spherical, the size function is isotropic and thus independent of the *hkl* indices. However, in the case of anisotropic crystallite shape the size function becomes anisotropic and *hkl* dependent, *cf*. (Langford et al., S1993). Now we calculate the size profile-function for *spherically ellipsoidal* shape and *lognormal* size distribution (Ribárik et al., S2001; Ribárik et al., S2004). The determination of the $g(M)\mathrm{d}M$ expression is done in the following steps taking into account the notations shown in Fig. 7.

1. Construction of the equation of the ellipsoid with ellipticity $\varepsilon=c/a$ in the eigensystem denoted by x', y', z'.
2. Transformation by rotation of the angle α around the axis x into the system x, y, z, where α is the angle between the diffraction vector and the revolution axis of the ellipsoid and z is parallel to **g**.
3. Expressing the column length M as $M(x, y)=z_2(x, y)-z_1(x, y)$, where z_1 and z_2 are determined by the equation of the ellipsoid.
4. Determination of the area, $T(M)$, of the curved plane given by the equation $M(x, y)=M$.
5. For one crystallite the following is obtained:

$$g(M)\mathrm{d}M = M[T(M)-T(M+\mathrm{d}M)] = \frac{\pi}{2}h(\varepsilon,\alpha)M^2\mathrm{d}M\,, \quad (43)$$

where $h(\varepsilon,\alpha)$ is given by the equation of $M(x, y)$. We note that in the case of a sphere: $h(1,\alpha)=1$.

6. Calculation of the maximum column length M_{max}:

$$M_{max} = \frac{2a}{\sqrt{1+\left(\frac{1}{\varepsilon^2}-1\right)\cos^2\alpha}}\,. \quad (44)$$

7. Summing up for all crystallites with the column length M and using the lognormal size distribution density function $f(x)$:

$$g(M)\,\mathrm{d}M \sim h(\varepsilon,\alpha)M^2\,\mathrm{erfc}\left[\frac{\log\left(\frac{M\sqrt{1+\left(\frac{1}{\varepsilon^2}-1\right)\cos^2\alpha}}{m}\right)}{\sqrt{2}\sigma}\right]\mathrm{d}M\,. \quad (45)$$

The size profile-function obtained in this way is identical to the one corresponding to spherical crystallites with the only difference that the m parameter becomes hkl-dependent:

$$m_{hkl} = \frac{m_A}{\sqrt{1+\left(\frac{1}{\varepsilon^2}-1\right)\cos^2\alpha_{hkl}}}\,, \quad (46)$$

where m_A is the now the m parameter of the size distribution. If the relative orientation between the unit cell vectors and the axis of revolution of the ellipsoid are known, $\cos\alpha_{hkl}$ can be expressed with the hkl indices. For cubic crystals, if the axis of revolution is parallel to the unit cell vector **a**:

$$\cos\alpha_{hkl} = \frac{l}{\sqrt{h^2+k^2+l^2}}\,. \quad (47)$$

For hexagonal crystals, if the axis of revolution is parallel to the unit cell vector **c**:

$$\cos\alpha_{hkl} = \frac{l}{\sqrt{\frac{4}{3}\frac{c^2}{a^2}(h^2+hk+k^2)+l^2}}\,. \quad (48)$$

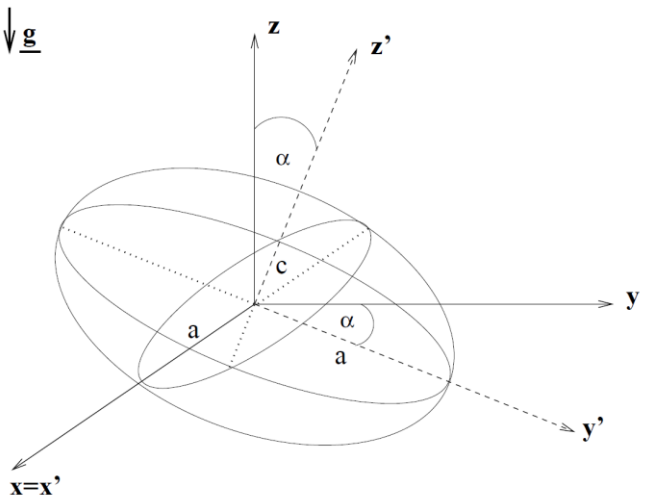

Figure 7. Notations used in the determination of the ellipsoidal size profile-function. The radii of the spherical ellipsoid are: *a* and *c*. There are two coordinate systems: *x*, *y*, *z* and *x*′, *y*′, *z*′, where latter is the eigensystem. The two coordinate systems are rotated by an angle of α around the *x* axis. The diffraction vector **g** is also indicated. The goal is to determine g(*M*)d*M* based on the geometrical properties of the part of the ellipsoid with column length between *M* and *M*+d*M* parallel to **g**.

We note that Scardi & Leoni (S2002) calculated the *hkl* dependent size broadening for different polyhedra, which is another possibility to introduce size anisotropy.

3. Strain broadening

In a real crystal, due to the lattice defects, the atoms are displaced relative to their ideal position. For this reason the reciprocal space may also be distorted and the condition of diffraction is satisfied not only in the ideal positions of the reciprocal lattice points, but in a finite volume in their proximity. This effect is called strain broadening. Warren (1959) gave the Fourier transform of the X-ray line profile if size and strain effects are present simultaneously:

$$A(L) = A^S(L)A^D(L) \;, \qquad (49)$$

where the strain Fourier coefficients can be expressed in the following form:

$$A^D_{hkl}(L) \cong \exp[-\, 2\pi^2 g^2 L^2 \langle \varepsilon^2_{g,L} \rangle] \;, \qquad (50)$$

where g is the absolute value of the diffraction vector, $\langle \varepsilon^2_{g,L} \rangle$ is the *mean square strain* (mss) depending on the displacement of the atoms relative to their ideal position and the brackets indicate spatial averaging. According to the continuum theory of elasticity, the longitudinal strain parallel to the direction of the **g** diffraction vector is defined as (Wilkens, 1970):

$$\varepsilon_{\mathbf{g}}(L,\mathbf{r}) = \frac{\frac{\mathbf{g}}{|\mathbf{g}|}\mathbf{u}(\mathbf{r}+\frac{L}{2}\frac{\mathbf{g}}{|\mathbf{g}|}) - \frac{\mathbf{g}}{|\mathbf{g}|}\mathbf{u}(\mathbf{r}-\frac{L}{2}\frac{\mathbf{g}}{|\mathbf{g}|})}{L}, \quad (51)$$

where **u** is the displacement field and L is the distance of the lattice points $\mathbf{r}+\frac{L}{2}\frac{\mathbf{g}}{|\mathbf{g}|}$ and $\mathbf{r}-\frac{L}{2}\frac{\mathbf{g}}{|\mathbf{g}|}$. With this the mss is obtained as:

$$\langle\varepsilon^2_{\mathbf{g},L}\rangle = \frac{\iiint_V (\varepsilon_{\mathbf{g}}(L,\mathbf{r}))^2\, \mathrm{d}^3\mathbf{r}}{\iiint_V \mathrm{d}^3\mathbf{r}}. \quad (52)$$

For discrete atoms, the strain, $\varepsilon_{\mathbf{g},L}$ is defined as:

$$\varepsilon^{(j,j')}_{\mathbf{g},n} = \frac{\frac{\mathbf{g}}{|\mathbf{g}|}\mathbf{u}(\mathbf{r}_j) - \frac{\mathbf{g}}{|\mathbf{g}|}\mathbf{u}(\mathbf{r}_{j'})}{nl_0}, \quad (53)$$

where j and j' are the indices of the atoms in the direction of **g**, $n = j - j'$, $L = nl_0$ and l_0 is the distance between two atoms in the **g** direction (Warren, 1959). The mss is obtained as the square of the strain averaged over all atomic pairs with the distance of nl_0:

$$\langle\varepsilon^2_{\mathbf{g},L}\rangle = \frac{\sum_{j-j'=n} (\varepsilon^{(j,j')}_{\mathbf{g},n})^2}{\sum_{j-j'=n} 1}. \quad (54)$$

Several authors worked on the determination of the mean square strain, including Warren (1959), Krivoglaz & Ryaboshapka (1963) and Wilkens (1970). Warren (1959) assumed either random atomic displacements and/or stacking faults. Krivoglaz (1969) and Wilkens (1970) assumed dislocations as the main source of strain broadening. Krivoglaz & Ryaboshapka (1963) assumed a totally random distribution of dislocations in the entire crystal and obtained the following expression for small L values:

$$\langle\varepsilon^2_{\mathbf{g},L}\rangle = \left(\frac{b}{2\pi}\right)^2 \pi\rho C \log\left(\frac{D}{L}\right), \quad (55)$$

where D is the crystal size, ρ is the dislocation density, C is the contrast factor of dislocations (see more details below) and b is the Burgers vector of dislocations. The problem with this formula is that (i) it diverges as D tends to infinity and (ii) using this strain function, the strain Fourier transform does not decay as L tends to infinity. For small L values, however, this logarithmic expression enables to estimate the dislocation density (Ribárik, 2008). The logarithmic behavior of the mss for small L values was derived by a rather exact method by Groma (1998, 2003), where it was shown that this is a general property of any dislocation configuration.

3.1. The Krivoglaz–Wilkens model of strain broadening caused by dislocations

There is a close correlation between strain broadening and the elastic stored energy of dislocations (Wilkens, S1969; Kocks & Scattergood, S1969; Groma, 1998). Based on that, Wilkens realized that the logarithmic singularity in eq. (55) is similar to the logarithmic singularity in the elastic stored energy of dislocations (Nabarro, S1952). In the case of the elastic stored energy of dislocations the logarithmic singularity is renormalized by introducing the effective outer cut-off radius of dislocations, R_e (Nabarro, S1952). The physical meaning of R_e is that the dislocations strain fields are screened in a dislocation ensemble and therefore do not reach out to longer distances than R_e from any arbitrary position within the ensemble. Wilkens improved the Krivoglaz model by replacing D by R_e^* in eq. (55). The restrictedly-random dislocation distribution was introduced, where the crystal is assumed to consist of separate regions with diameters of R_e^*. Each R_e^* diameter region consists of pairs of plus and minus straight parallel screw dislocations of the density ρ distributed randomly within these regions. There is no interaction between dislocations outside of these regions. Wilkens (1970) derived the mss in the entire *L* range:

$$\langle \varepsilon_{\mathbf{g},L}^2 \rangle = \left(\frac{b}{2\pi}\right)^2 \pi\rho C f\left(\frac{L}{R_e^*}\right), \qquad (56)$$

where *f* is the strain function. In the following we call *f* the Wilkens function. It was developed numerically in the entire *L* range given in eqs. (A6-A8) in Appendix-A in Wilkens (1970):

$$f^*(\eta) = -\log\eta + \left(\frac{7}{4} - \log 2\right) + \frac{512}{90\pi}\frac{1}{\eta} +$$

$$\frac{2}{\pi}\left[1 - \frac{1}{4\eta^2}\right]\int_0^\eta \frac{\arcsin V}{V}\mathrm{d}V -$$

$$\frac{1}{\pi}\left[\frac{769}{180}\frac{1}{\eta} + \frac{41}{90}\eta + \frac{2}{90}\eta^3\right]\sqrt{1-\eta^2} -$$

$$\frac{1}{\pi}\left[\frac{11}{12}\frac{1}{\eta^2} + \frac{7}{2} + \frac{1}{3}\eta^2\right]\arcsin\eta + \frac{1}{6}\eta^2, \quad \text{if } \eta \le 1,$$

$$f^*(\eta) = \frac{512}{90\pi}\frac{1}{\eta} - \left[\frac{11}{24} + \frac{1}{4}\log 2\eta\right]\frac{1}{\eta^2}, \quad \text{if } \eta \ge 1, \qquad (57)$$

where $f\left(\frac{L}{R_e^*}\right) = f(\eta^*)$ and $\eta = \frac{1}{2}\exp\left(-\frac{1}{4}\right)\frac{L}{R_e^*}$. The Wilkens function and its approximations for small and large values of *L* are plotted in Fig. 8. In the Wilkens function the same logarithmic term is present as in the Krivoglaz model, but it does not diverge with the crystallite size, since it depends on the correlation-length parameter, R_e^*, renormalizing the logarithmic singularity in eq. (55). Both the Krivoglaz and Wilkens models reveal a logarithmic singularity at small *L* values. This, however, has no significant effect on the shape of line profiles considerably because in the Fourier transform of the strain profiles, $A^D(L)$ the mss is multiplied by L^2 compensating this

singularity to a great extent: $L^2\log L\rightarrow 0$, if $L\rightarrow 0$. We note that in most practical cases instead of R_e^* the effective outer cut-off radius $R_e=R_e^*\exp(2)$ is used. The reason for this is that in the paper of Wilkens (1970) R_e^* was used for easing calculus-technical processes. However, the physical quantity related to true distances is R_e (Ungár et al. 1984; Wilkens, 1988; Hecker et al., 1997).

Inserting eq. (56) into (50) the Fourier–transform of the strain profile is obtained:

$$A^D(L) = \exp\left[-\frac{\pi b^2}{2}(g^2C)\rho L^2 f\left(\frac{L}{R_e^*}\right)\right] . \qquad (58)$$

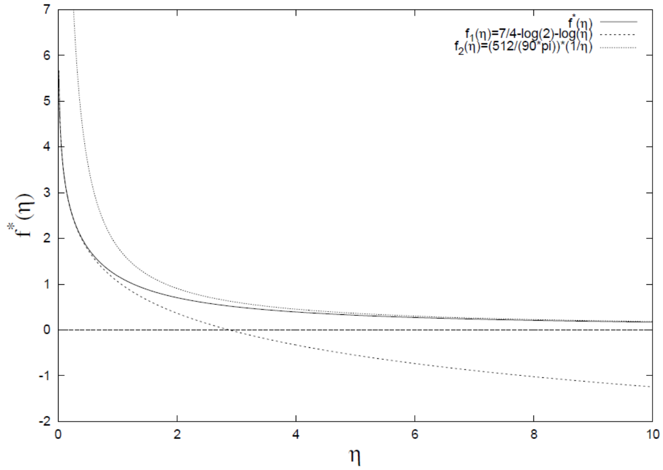

Figure 8. The $f^*(\eta)$ Wilkens function (solid line) and the two approximations. The dash-line curve is the logarithmic part at small L values. In the figure it is approximates as: $f_1(\eta)=\frac{7}{4}-\log 2-\log(\eta)$. The dotted-line curve is the hyperbolic part at large L values approximated as: $f_2(\eta)=\frac{512}{90\pi}\,\frac{1}{\eta}$. Using numerical simulations Kamminga and Delhez (S2000) showed that the strain profile calculated by the Wilkens-model is also valid for edge dislocations and curved dislocations.

The actual value of R_e^* or R_e depends on the actual value of the dislocation density, ρ. In order to avoid this ambiguity Wilkens (1970) introduced the dimension free *dislocation arrangement* parameter:

$$M^* = R_e^*\sqrt{\rho} \text{ or } M=R_e\sqrt{\rho} . \qquad (59)$$

The value of M^* or M is large when the dislocations are arranged uncorrelated randomly and the related strain fields are of long-range character. On the contrary, M^* or M is small when opposite sign dislocations are in strong correlation close to each other and the related strain fields, due to screening, are of short range character. A schematic illustration is shown in in Fig. 9, where 12 dislocations are randomly distributed in Fig. 9a and in strong dipole configuration in Fig. 9b. In the case of random distribution the strain fields reach out much further than the average

dislocation distance, d_{disloc}, whereas in the case of strong dipole configuration the strain fields are strongly screened and R_e becomes shorter than d_{disloc}. The M value is in direct correlation with the average dislocation distance:

$$M = R_e\sqrt{\rho} = \frac{R_e}{d_{disloc}}\ , \tag{60}$$

The random or the strong correlated dislocation arrangements can be called weak or strong dipole character. In the first case M is larger than unity, $M \gg 1$, whereas in the second case it is close to unity or even smaller: $M \leq 1$.

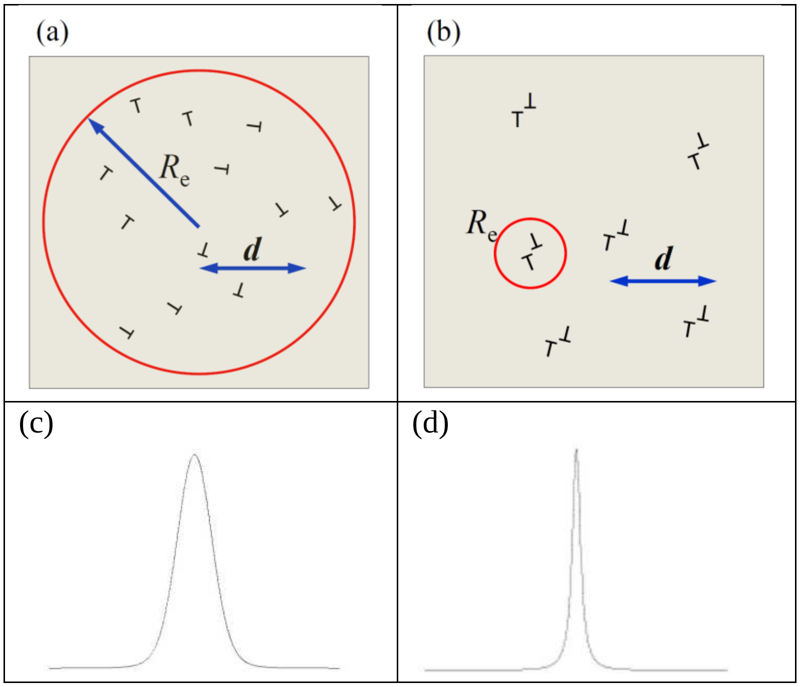

Figure 9. A schematic interpretation of the dislocation dipole character parameter, M=$R_e\rho$. (a) Random dislocation distribution, where M⟩⟩1. (b) Dislocations arranged in strong dipole configuration where M⟨⟨1. (The same type of figures can be drawn with R_e^*.) (c) and (d) Schematic strain profiles corresponding to the dislocation arrangements in (a) and (b), respectively. In the case of (a) and (c) $R_e \rangle\rangle 1/\sqrt{\rho}$ and M⟩⟩1, whereas in the case of (b) and (d) $R_e \langle\langle 1/\sqrt{\rho}$ and M≤1. (The *y* scale of the profiles in (c) and (d) is logarithmic.)

Due to the reciprocity of length scales in crystal and reciprocal space, when the strain fields are of long or short range character the tail regions of diffraction peaks decay faster or slower and the related M or M* parameters are large or small, respectively. Fig. 10 shows strain profiles for the same dislocation density, ρ, but for different M^* values.

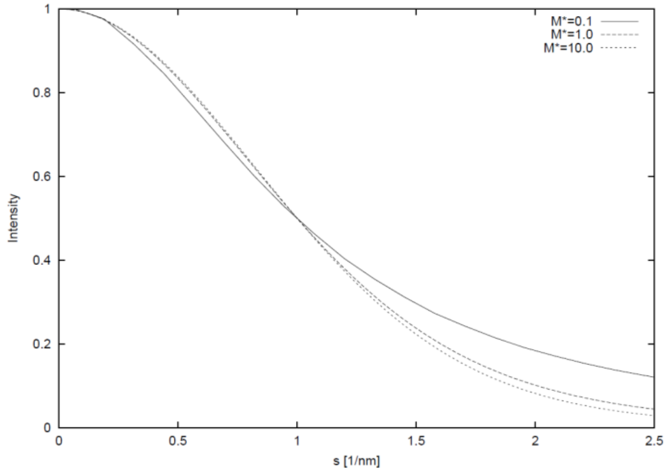

Figure 10. The shape of strain profiles for fixed ρ and variable *M** values. Note that *s* is normalized by the FWHM values of the profiles.

The Fourier transform of the strain profile given by the Wilkens model is real, therefore its inverse Fourier transform, which is the strain profile itself, is symmetrical. Strain profiles are, however, not necessarily symmetrical. For example in the case of tensile deformed Cu single crystals (Ungár et al., 1984; Mughrabi et al., 1986; Gaál, 1973, 1976, 1984; Jakobsen et al., 2006; Jakobsen et al., 2007) or in tensile deformed lath martensitic steels (Ungár et al., 2017; Harjo et al, 2017; Ódor et al., 2020) strain profiles are characteristically asymmetric. By characteristically asymmetry we mean that the peak asymmetry is of the opposite sense in patterns measured on planes normal (axial-case) or parallel (side-case) to the tensile direction. Characteristic peak asymmetry was explained by the composite model of heterogeneous microstructures developed by Mughrabi et al. (1986) for dislocation cell structures. A more fundamental theoretical description was given by Groma (1998) taking into account dislocation-dislocation correlation and the formation of polarized dislocation dipoles.

3.2. Strain anisotropy: the concept of contrast factors

Strain anisotropy means that the broadening of the profiles show an anisotropic behavior as a function of the *hkl* indices: the width of the profiles is not a monotonous function of the length of the diffraction vector or its square, see for example the Williamson-Hall type plot (Williamson & Hall, 1953) of ball milled WC (Gillies & Lewis, S1968) in Fig. 11 or plastically deformed copper in Fig. 12a. Dislocations are extremely anisotropic lattice defects giving contrast in a diffraction experiment only under specific diffraction conditions. In TEM the visibility criterion of dislocations is **gb**≠0. The schematic drawing in Fig. 11 shows the same dislocation in two different diffraction conditions. One, (a), where **g** and **b** are parallel and the curved reflecting lattice planes are expected to cause large strain broadening. The other, (b), where **g** and **b** are normal to each other and the non-affected straight reflecting planes leave diffraction peaks narrow.

Line broadening can be visualized by the FWHM or the integral breadths, β (both in reciprocal space coordinates, K=d*=1/d) *vs*. K (Stokes & Wilson, 1944; Hall, 1949; Williamson & Hall, 1953):

FWHM=$\frac{0.9}{D}$ + εK , β=$\frac{1}{D}$ + εK , (61)

where D and ε are related to the crystallite size and the microstrain. Line broadening is assumed

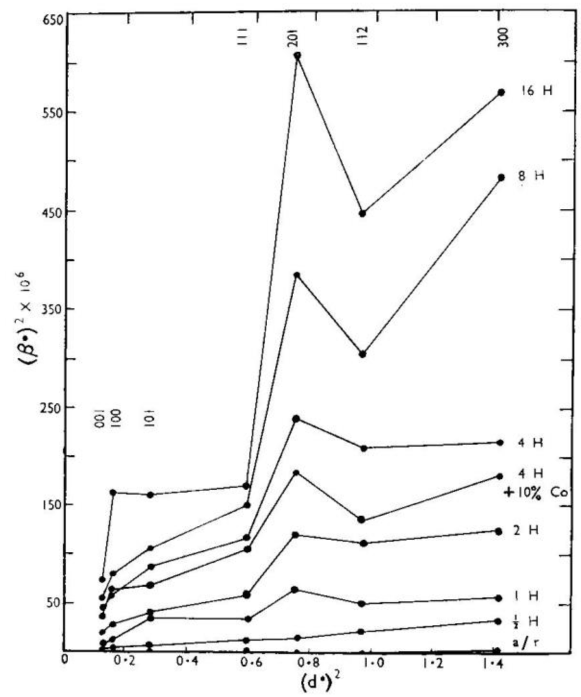

Figure 11. The squared integral breadth, β *vs*. the squared d* of WC X-ray diffraction patterns. WC was ball-milled to different periods of times up to 16 h (Gillies & Lewis, 1968).

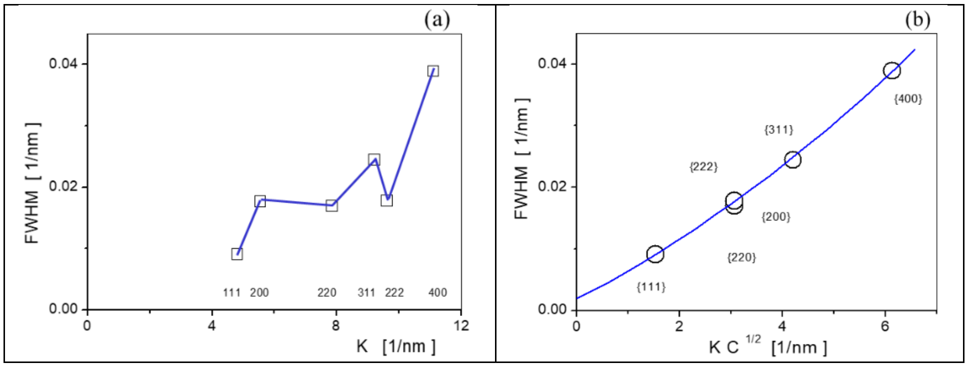

Figure 12. (a) The FWHM *vs*. K=d* of Cu X-ray diffraction patterns. The specimen was deformed by equal-channel-angular-pressing (ECAP). (b) The same FWHM values as in (a) *vs*. K$\sqrt{\bar{C}}$, where $\bar{C}$ is the average contrast factor of Cu (Ungár & Borbély, 1996).

to be caused by microstrain whose spatial average is zero. Line broadening does not change lattice spacings. Therefore it is more appropriate to use $\sqrt{\langle \varepsilon_{g,L}^2 \rangle}$ instead of ε in eq. (14):

$$\mathrm{FWHM}=\frac{0.9}{D}+\sqrt{\langle\varepsilon_{g,L}^{2}\rangle}\mathrm{K}\ ,\quad \beta=\frac{1}{D}+\sqrt{\langle\varepsilon_{g,L}^{2}\rangle}\mathrm{K}\ . \tag{62}$$

In a texture free polycrystalline sample, an ideal powder specimen or in a single crystal where all possible slip systems are equally populated the contrast factor has to be isotropic for a particular *hkl* reflection. This means that the individual contrast factors, *C* have to be averaged over all possible permutations of *hkl* (Ungár & Tichy, 1999).

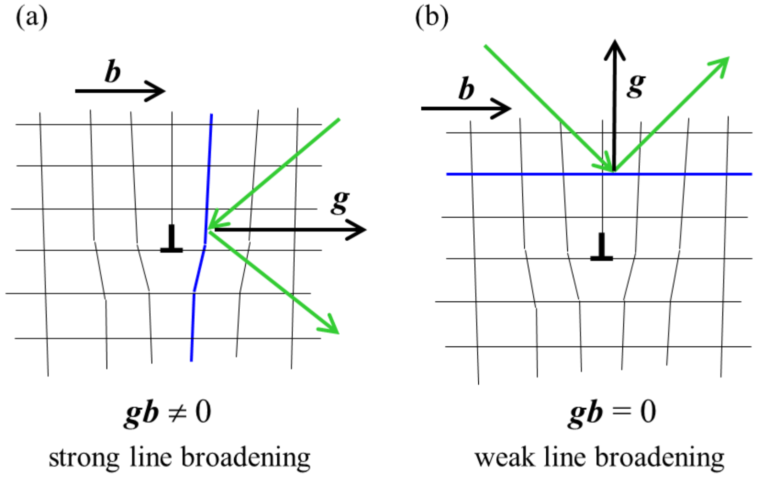

Figure 13. The two schematic drawings (a) and (b) show the same edge dislocation (symbolized by the reversed T) with the lattice plane traces around them. The green arrow lines indicate in and outgoing radiation beams reflected on the blue highlighted planes. In (a) the reflecting planes are curved and **gb**≠0, thus strong strain broadening is occurring. In (b) the reflecting planes are unaffected and **gb**=0, in this case no strain broadening is occurring (this is true only schematically).

The longitudinal strain parallel to the diffraction vector $\boldsymbol{g}$ is [25]:

$$\varepsilon_g = \frac{\partial}{\partial\xi}(\boldsymbol{gu})/g\ , \tag{63}$$

where $\boldsymbol{u}$ is the displacement field of dislocations and ξ is the direction parallel to $\boldsymbol{g}$. On the right hand side of equation (56) the only *hkl* dependent term is *C*. Tus the *hkl* dependence of *C* can be obtained from the expression:

$$C \propto <[\frac{\partial}{\partial\xi}(\boldsymbol{gu})/g]^2> = \left\langle\left(\frac{\boldsymbol{g\beta g}}{g^2}\right)^2\right\rangle\ , \tag{64}$$

where $\boldsymbol{\beta}=grad\mathbf{u}$ is the distortion tensor. The pointed brackets indicate averaging over the crystal volume. Relation (64) shows that Cg^4 is a fourth order polynomial of the *hkl* indices:

$$Cg^4 = P_4(hkl) \tag{65}$$

The average values of the contrast factors, $\overline{C}$, is obtained by averaging $P_4(hkl)$ over all possible permutations of the *hkl* indices of any particular reflection. This means that we have to find the invariants of P_4 with the requirement that they satisfy the symmetry of the particular crystal structure. For cubic crystals the only fourth order invariant except the trivial g^4 is:

$$h^2k^2 + h^2l^2 + k^2l^2 \ . \tag{66}$$

With this

$$\overline{C} = \frac{P_4}{g^4} = A + B\,\frac{h^2k^2 + h^2l^2 + k^2l^2}{\left(h^2 + k^2 + l^2\right)^2}\ , \tag{67}$$

where *A* and *B* are constants. Using the notation:

$$\mathrm{H}^2 = \frac{h^2k^2 + h^2l^2 + k^2l^2}{\left(h^2 + k^2 + l^2\right)^2}\ , \tag{68}$$

the average contrast factors of dislocations for cubic crystals will be:

$$\overline{C} = A + B\mathrm{H}^2 \ . \tag{69}$$

The constants *A* and *B* depend on the elastic constants of the crystal and the type of dislocations, e. g. edge and/or screw character (Ungár et al., 1999; Borbély et al., 2003; Martinez-Garcia et al., 2008). If *B* is not zero eq (69) can be given as:

$$\overline{C} = \overline{C}_{h00}(1-qH^2)\ , \tag{70}$$

where $\bar{C}_{h00}$ is the average contrast factor of the *h00* reflections and *q* is a constant depending on the elastic constants of the crystal and the dislocation type. The *q* parameter is shown in Fig. 14 for edge and screw type dislocations in *fcc* and *bcc* crystals as a function of the elastic anisotropy, A_i, of crystal (Ungár et al., 1999). Note that for <*h00*> type Burger vectors *B* is zero, therefore in this case only eq. (69) is valid. In the case of a particular specimen at the corresponding A_i anisotropy parameter value (where $A_i=2c_{44}/(c_{11}-c_{12})$ and c_{11}, c_{12}, and c_{44} are the elastic constants of the material) there is a window for the *q* parameter between the q_{edge} and q_{screw} values belonging to edge or screw character dislocations. From the experimentally determined *q* parameter value the fraction of edge or screw character dislocations, f_{edge} or f_{screw}, can be determined:

$$f_{\text{edge}} = \frac{q - f_{edge}}{f_{screw} - f_{edge}}\ ,\ f_{\text{screw}} = \frac{f_{screw} - q}{f_{screw} - f_{edge}}\ . \qquad (71)$$

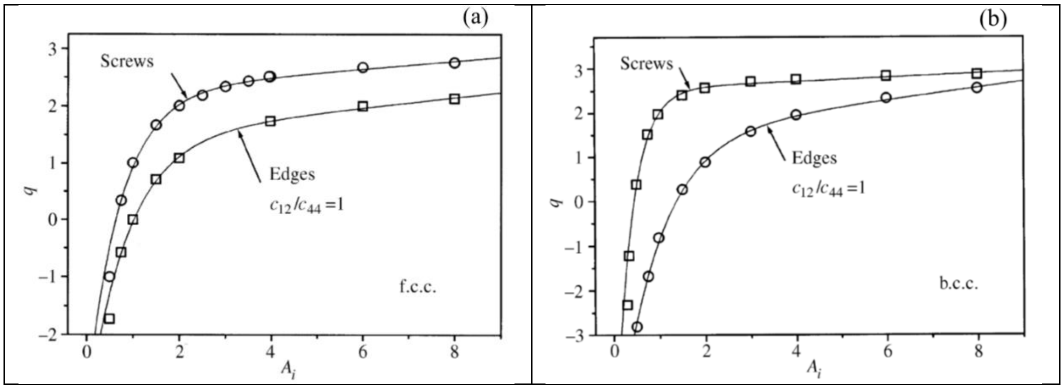

Figure 14. The q parameter *vs*. the elastic anisotropy A_{i} and the ratio of the elastic constants c_{12}/c_{44}: (a) in the case of *fcc* screw and edge dislocations, the latter when c_{12}/c_{44}=1; (b) in the case of *bcc* screw and edge dislocations, the latter when c_{12}/c_{44}=1.

For hexagonal crystals the average contrast factors are (Ungár & Tichy, 1999; Dragomir & Ungár, 2002):

$$\bar{C} = \bar{C}_{hk.0}(1 + a_1 H_1^2 + a_2 H_2^2)\ , \qquad (72)$$

where

$$H_1^2 = \frac{[h^2+k^2+(h+k)^2]l^2}{\left[h^2+k^2+(h+k)^2+\frac{3}{2}\left(\frac{a}{c}\right)^2 l^2\right]^2}\ ,\quad H_1^2 = \frac{l^4}{\left[h^2+k^2+(h+k)^2+\frac{3}{2}\left(\frac{a}{c}\right)^2 l^2\right]^2}$$

and a and c are the lattice constants of the *hcp* crystal.

For orthorhombic crystals the average contrast factors will be (Ungár & Tichy, 1999; Ribárik, 2008):

$$\bar{C} = \bar{C}_{h00}(H_0^2 + a_1 H_1^2 + a_2 H_2^2 + a_3 H_3^2 + a_4 H_4^2 + a_5 H_5^2)\ , \qquad (73)$$

where

$$H_0^2 = \frac{\frac{h^4}{a^4}}{\left(\frac{h^2}{a^2}+\frac{k^2}{b^2}+\frac{l^2}{c^2}\right)^2},\quad H_2^2 = \frac{\frac{l^4}{c^4}}{\left(\frac{h^2}{a^2}+\frac{k^2}{b^2}+\frac{l^2}{c^2}\right)^2},\ H_4^2 = \frac{\frac{l^2h^2}{c^2a^2}}{\left(\frac{h^2}{a^2}+\frac{k^2}{b^2}+\frac{l^2}{c^2}\right)^2},$$

$$H_1^2 = \frac{\frac{k^4}{b^4}}{\left(\frac{h^2}{a^2}+\frac{k^2}{b^2}+\frac{l^2}{c^2}\right)^2},\quad H_3^2 = \frac{\frac{h^2k^2}{a^2b^2}}{\left(\frac{h^2}{a^2}+\frac{k^2}{b^2}+\frac{l^2}{c^2}\right)^2},\ H_5^2 = \frac{\frac{k^2l^2}{b^2c^2}}{\left(\frac{h^2}{a^2}+\frac{k^2}{b^2}+\frac{l^2}{c^2}\right)^2}. \qquad (74)$$

The constants C_{h00} and $C_{hk.0}$ are calculated on the basis of the crystallography of dislocations and from the elastic constants of the crystal (Ungár et al, 1999). The q, a_1,a_2, . . . and a_5 parameters are global for all reflections in a particular diffraction pattern. They are related to slip modes and edge or screw dislocation character. Borbély et al. (2003) developed a freely available numerical procedure, ABIZC, for the calculation of individual or average contrast factors taking into account the elastic constants of the material, the lattice parameters and the relative orientations of the **l**, **b**, **n** and **g** vectors.

The CMWP procedure provides the **weighted sum of squared residual** (WSSR) and the **goodness of fit** (GoF) at the end of the fitting procedure listed in the *.sol file.

References (Supplement)

Aitchinson, J. and Brown, J., *The Lognormal Distribution*, Cambridge University Press, Cambridge (1957)

Arley, N. and Buch, K. R., *Introduction to the Theory of Probability and Statistics*, New York - Wiley, (1950)

Bertaut, F. C. R., *Acad. Sci., Paris*, **228**, 187 (1949)

Borbély, A., Dragomir, I. C., Ribárik, G, Ungár, T., Computer program ANIZC for the calculation of diffraction contrast factors of dislocations in elastically anisotropic cubic, hexagonal and trigonal crystals, *J. Appl. Cryst*., **36**, 160–162, (2003)

Gaál, I., Acta Phys. Acad. Sci. Hung. 33, 411–418 (1973)

Gaál, I., Dissertation to the Hungarian Academy of Sciences, Budapest, Hungary (1976)

Gaál, I., Proc. 5th Riso Int. Syrup. Metall. Mater. Sci., edited by N. H. Andersen, M. Eldrup, N. Hansen, D. Juul Jensen, T. Leffers, H. Lifholt, O. B. Pedersen B. N. Singh, pp. 249-254. Riso Natl Lab., Roskilde, Denmark (1984)

Gillies, D.C., Lewis, D., (1968) "Lattice Strain in Tungsten Carbide", *Powder Metallurgy*, **11**, 400-410.

Groma, I., X-ray line broadening due to an inhomogeneous dislocation distribution, *Phys. Rev*. B, 57 (1998) 7535–7542.

Guinier, A., *X-ray diffraction*, Freeman and Co., San Francisco, CA (1963)

Hall, W. H., Proc. Phys. Soc., A62 (1949) 741.

Hinds,W. C., *Aerosol Technology: Properties, Behavior and Measurement of Airbone Particles*, Wiley, New York (1982)

Jakobsen B, Poulsen HF, Lienert U, Almer J, Shastri SD, Sørensen HO, Gundlach C, Pantleon W., "Formation and subdivision of deformation structures during plastic deformation" *Science*, 2006 312, 889-892.

Jakobsen B, Poulsen HF, Lienert U, Pantleon W., (2007) "Direct determination of elastic strains and dislocation densities in individual subgrains in deformation structures", *Acta Mater*., **55**, 3421-3430.

Kamminga, J. D. and Delhez, R., *J. Appl. Cryst.*, **33**, 1122–1127 (2000)

Krill, C. E. and Birringer, R., *Philos. Mag. A*, **77**, 621–640 (1998)

Kocks, U.F., Scattergood, R.O., Elastic Interactions Between Dislocations in a Finite Body, *Acta Metall.*, **1969**, *17, 1161-1168.*

Langford, J. I. and Wilson, A. J. C., J. Appl. Cryst., 11, 102–113 (1978)

Langford, J. I., Boultif, A., Auffrédic, J. P., and Louër, D. (1993). “The use of pattern decomposition to study the combined X-ray diffraction effects of crystallite size and stacking faults in ex-oxalate zinc oxide,” J. Appl. Crystallogr. 26, 22–33.

Langford, J. I., Louër, D. and Scardi, P., *J. Appl. Cryst.*, **33**, 964–974 (2000)

Martinez-Garcia, Leoni, M., Scardi, P., (2008) "A general approach for determining the diffraction contrast factor of straight-line dislocations", *Acta Cryst.*, **A65**, *109-119*.

Mughrabi H., Ungár T., Kienle W., Wilkens M., *Phil. Mag.*, **53**, 793 (1986)

Nabarro, F.,R.,N., (1952) "The Mathematical Theory of Stationary Dislocations", *Adv. Phys*., **1**, 269-395.

É, Ódor, B. Jóni, G. Ribárik, N. Q. Chinh, T. Ungár P. J. Szabó, (2020) "Deformation induced soft and hard lath packets enhance ductility in martensitic steels", *Crystals, in the press*.

Ribárik, G., Ungár, T. and Gubicza, J., MWP-fit: a program for multiple whole-profile fitting of diffraction peak profiles by ab initio theoretical functions, *J. Appl. Cryst.*, **34**, 669–676 (2001)

Ribárik, G., Gubicza, J. and Ungár, T., Correlation between strength and microstructure of ball milled Al-Mg alloys determined by X-ray diffraction, *Mat. Sci. Eng. A*, **A387–389**, 343–347 (2004)

Ribárik, G., Jóni, B. & Ungár, T. (2020) *Crystals*, The Convolutional Multiple Whole Profile (CMWP) Fitting Method, a Global Optimization Procedure for Microstructure Determination (2020) 10, 623; doi:10.3390/cryst10070623

Scherrer, P., *Göttinger Nachrichten*; R. Zsigmondy, Kolloidchemie (3rd Ed. 1920), pp. 394 (1918)

Scardi, P. and Leoni, M., *Acta Cryst.*, **A58**, 190–200 (2002)

Stokes, A. R., Wilson, A. J. C., Proc. Phys. Soc. 56 (1944) 174.

Terwilliger, Ch. D. and Chiang, Y. M., *Acta Metall. Mater.*, **43**, 319–328 (1995)

Ungár, T., Mughrabi, H., Roennpagel, D. & Wilkens, M. (1984). *Acta Metall*. **32**, 333-342.

Ungár, T., Dragomir, I. C., Révész, Á. and Borbély, A., *J. Appl. Cryst.*, **32**, 992–1002 (1999)

Ungár, T., Tichy, *Phys. Status Solidi*. A, 171, 425–434 (1999)

Ungár, T., Gubicza, J., Ribárik, G. and Borbély, A., Crystallite size-distribution and dislocation structure determined by diffraction profile analysis: Principles and practical application to cubic and hexagonal crystals, *J. Appl. Cryst.*, **34**, 298–310 (2001)

Ungár, T., Tichy, G., Gubicza, J., Hellmig, R. J., (S2005), Correlation between subgrains and coherently scattering domains, *J. Powder Diffr*., **20**, 366-375.

Valiev, R. Z., Kozlov, E. V., Ivanov, Yu., F., Lian, J., Nazarov, A. A. and Baudelet, B., *Acta Metall. Mater.*, **42**, 2467–2476 (1994)

Wilkens, M., Das Mittlere Spannungquadrat $<\sigma^2>$ Begrentzt Regellos Verteilter Vesetzungen in einem Zylinderförmigen Körper, *Acta Metall.*, **1969**, *17, 1155-1159.*

Williamson, G. K. and Hall, W. H., *Acta metall.*, **1**, 22–31 (1953)

Zilahi, Gy., Ungár, T., Tichy, G., (2015) "A common theory of line broadening and rocking curves" *J. Appl. Cryst.*, **48**, 418–460.

York, B. R., *Adv. X-ray Anal.*, **41**, 544–555 (1999)
