# Supplementary material for: Characterizing dislocation loops in irradiated polycrystalline Zr alloys by X-ray line profile analysis of powder diffraction patterns with satellites
Source: J Appl Crystallogr. 2021 May 25;54(Pt 3):803–21. doi: 10.1107/S1600576721002673 (PMC8202032; doi:10.1107/S1600576721002673)
Supplement: Supplementary file 2 [file j-54-00803-sup2.pdf]

Supplement S2

Manual of the JAVA frontend of CMWP

2020

Gábor Ribárik and Tamás Ungár

This brief manual of the CMWP line-profile-analysis procedure describes the frontend usage. The principles and detailed structure of CMWP is given in the references at the end of this manual.

All lengths are given in units of nanometer [nm].

The items (usually in bold below) appear in the JAVA frontend shown in the "Frontend images" Supplement, S5.

CUBIC, HEXAGONAL, ORTHOROMBIC

Click the appropriate crystal structure.

lat_a, lat_b, lat_c,
Put in the corresponding lattice constants,
for cubic or hexagonal leave lat_b and lat_c, or lat_b empty, respectively.

Burgers vector
Here you can insert the Burgers vector of dislocations,
for hexagonals or orthorombics this will be a "formal" values which will have to be "re-evaluated" taking into account the results of the contrast factors, once the patterns have been optimized.

Wavelength
Here you can insert the wavelength you are using.
When neutron diffraction patterns are worked on you have the option to use d*=1/d=K.
In such a case you have to click: "Fit in K instead of 2*theta".
Despite that in such a case the "wavelength" value will not be used, the "wavelength" window must be filled in.

Ch00 or Chk0:
When average dislocation contrast factors can be used you can insert the
$\acute{C}_{h00}$, or $\acute{C}_{hk0}$ values for cubic or hexagonal crystals, respectively.
For more details see: (Ungár *et al.*, 1999; Dragomir & Ungár, 2002; Dragomir & Ungár, 2002a; Ribárik, 2008)

Don't include size effect:
If you are sure that in your pattern there is no size-effect, you may click here,

however, it is better to set "init_b" to 10 and "init_c" to 1, and "fix" these two values, see more to these two parameters below.

Use ellipsoidal size func.:
If you suspect that your coherently-scattering-domains are better approached as "rotational ellipsoids" than spheres you may click here.
In such a case the "init-epsilon" becomes active.

Use individual C factors:
When you suspect that for any reason the average dislocation contrast factors are not appropriate, you may click here, in such a case the values in "Ch00" or "Chk0" become irrelevant; when this option is activated the value of "init_d" will not be changed and the dislocation density provided in this case can only be considered as a "formal" value; for more details see: (Cordier *et al*., 2004; Ungár *et al*., 2010; Balogh *et al*., 2010; Ribárik, 2008)

Include St.Fault effect:
If you suspect or have indications that your specimen does contain planar defects, i.e. twin boundaries, or other kind of planar defects, like intrinsic or extrinsic stacking-faults, you can click here. At the same time you have to connect the appropriate "stacking.dat file" by using the "Browse" button. The "stacking.dat file"-s are free to download using the CMWP homepage. For details see: (Balogh *et al*., 2006; Balogh *et al*., 2009)

stacking, dat file
See the item before.

Use weights:
When there are very large differences in the intensity-maxima of the different reflections, e.g. of the order of 10^2 or 10^3, you may click here, the peaks will be weighted, within a certain 2θ or d* range, by the reciprocal of the square of the intensity maxima of the profiles. You have to bare in mind that weighting will also change the emphasis of the different peaks as the contribution to the entire evaluation. The effect of weighting can be assessed by inspection of the fitting quality by using the "View FIT" button.

Disable coinc. g^2 code:
when working on single-crystals you may have profiles with the same d* values but corresponding to different orientations. In this case you can evaluate these profiles simultaneously, however, you must apply this button, along with this it is advisable to apply the "Use individual C factors:" option.

Use instrum. profiles:
for instrumental corrections you have to apply this button. At the same time you have to include the directory consisting of the appropriately prepared instrumental profiles. For this you have to use the "Browse" for the "Instrum.profile dir" position, for more details see the manual of CMWP or the PhD-work of Ribarik (2008), both free to download from the CMWP homepage.

Fit peak int.:, Fit peak pos.:
The CMWP procedure is not refining the unit cell, therefore, both the peak intensities and the peak positions are "subsidiary" parameters in the entire optimization procedure. Since the initially determined peak intensities and positions may not have been the ideal values for optimizing the

physical profiles functions and physical parameters, both the peak intensities and the peak positions can be adjusted by clicking these two buttons. The two can be activated simultaneously or sequentially according to your own decision.

Fit in K instead of 2*theta:
If the pattern is given in $K=2\sin\theta/\lambda=d^*=1/d$, this button has to be activated. This option is especially useful for neutron diffraction patterns measured in TOF mode.

Clone peak-index.dat file:
When a series of patterns are evaluated where the peak positions and intensities do not change considerably this button can be used to import already existing peak-index.dat files.

Clone bg-spline.dat file:
When a series of patterns are evaluated where the background does not change considerably, this button can be used to import already existing bg-spline.dat files.

FT limit (if no instr.eff.):
When the instrumental effect is negligible and there is no need for instrumental correction the smallest value of the FT has to be limited. The default value is 10^{-7}.

Profile cutting parameter:
The theoretical profile functions will be calculated for the first indexed Bragg reflection in the $\Delta K=2[\sin(\theta+\theta_0)/\lambda- \sin(\theta-\theta_0)/\lambda]$ range, where $2\theta_0$ is the Bragg angle of the first indexed Bragg reflections and $\theta-\theta_0$ is the value of the cutting parameter. All higher angle reflections will be evaluated within the same ΔK range; the appropriate value of the "cutting parameter" depends on the broadening of peak profiles; it should be large enough in order to include the "tail" parts into the evaluation, however, if real "background" is involved into the evaluation the iterations may become somewhat longer.

N1; N2:
These are the numbers of sampling within the range determined by the "cutting parameter" and in-between, respectively. N2 is the sampling number in the background region throughout the entire pattern. The default is N1=N2=1024. Sometimes it might be useful to change this number to 512, especially if the software does not start working despite no obvious error.

Min. 2*theta/K, Max. 2*theta/K:
These are the 2θ or K range values of the pattern. Optimization is carried out within these two given minumum and maximum limits irrespective how long the range is in the *.dat file. It is, however advisable to adjust the *.dat file range to these limiting values. If the two windows for these two values are left black at the beginning, the software will automatically put in the smallest and largest 2θ or K vaues from the *.dat file. The gnuplot figures will be displayed in this range during the evaluation procedure.

init_a(CUB):
This is the q parameter in the average dislocation contrast factors for cubic crystals, if "CUBIC" is active at the top of the frontend. For starting the procedure there must be a number here, for details see (Ungár & Tichy, 1999).

init_a1(HEX|ORT), init_a2(HEX|ORT):
These are the a_1 and a_2 parameters in the average dislocation contrast factors for hexagonal or orthorhombic crystals. If "HEXAGONAL" is active at the top of the frontend, for starting the procedure there must be numbers here, for details see (Ungár & Tichy, 1999; Dragomir & Ungár, 2002; Ribárik, 2008)

a_fixed:, a1_fixed:, a2_fixed:
If the pattern is not good enough to provide physically reasonable values you may fix the values of these parameters to physically more appropriate ones. After optimizing the other physical parameters you can restarts with releasing the fixed parameters. In most cases such a strategy finally provides the global optimum of all physical parameter values.

init_a3(ORT), init_a4(ORT), init_a5(ORT):
These are the a_3, a_4, and a_5 parameters in the average dislocation contrast factors for orthorhombic crystals, if "ORTORHOMBIC" is active at the top of the frontend. For starting the procedure there must be numbers here, for details see (Ribárik, 2008).

a3_fixed:, a4_fixed:, a5_fixed:
If the pattern is not good enough to provide physically reasonable values for these parameters you may fix the values to ones you think are more appropriate. The other physically relevant parameters can still be determined.

init_epsilon:
If the size of the "coherently scattering domains" are anisotropic in specific crystallographic directions, you may click here, and your size broadening will be evaluated by assuming that the "coherently scattering domains" are rotational ellipsoids in specific crystallographic directions, the default value here is l, meaning shape-isotropy, for more details see the *phd-work of Ribarik* (Ribárik, 2008).

epsilon_fixed:
If necessary the initial value of "epsilon" can be fixed. In this case it will not be changed during the fitting procedure.

init_b:
This is the median, i.e. the m parameter, in the log-normal size distribution function related to the coherently scattering domain.

b_fixed:
If necessary the initial value of "b" can be fixed. In this case it will not be changed during the fitting procedure.

init_c:
This is the variance, i.e. the σ parameter, in the log-normal size distribution function related to the coherently scattering domain. A variety of average size-values can be obtained from m and σ as described in more detail in Ribárik et al. (2020).

c_fixed:
If necessary the initial value of "c" can be fixed. In this case it will not be changed during the fitting procedure.

init_d:
"d" is the dislocation density in 10^{14} m^{-2}.

d_fixed:
If necessary the initial value of "d" can be fixed. In this case it will not be changed during the fitting procedure.

init_e:
"e" is the effective outer cut-off radius of dislocations, R_e . The dislocation arrangemenet parameter is: $M^*=R_e \sqrt{\;}$. $M=e^2M^*$.

e_fixed:
If necessary the initial value of "e" can be fixed. In this case it will not be changed during the fitting procedure.

init_st_pr.:
This is the initial value for planar defect density in percentage. When activating this option the "stacking.dat file" must be filled up appropriately.

stacking_pr_fixed:
If necessary the initial value of " init_st_pr" can be fixed. In this case it will not be changed during the fitting procedure.

Rc:
The role of Rc is to limit the value of Re below a user edited value.

rhoIGS:
Intergranular elastic strains (IGS) are homogeneous with infinitely long correlation lengths. The corresponding mss is a constant. Formally, $\langle \; {}^{2}_{g,L} \rangle$=constant means that the profile function is of Gaussian shape. Since the profile shapes generated by dislocations and IGSs are so different it is assumed that the mss can be written as:

$$\langle \; {}^{2}_{g,L} \rangle = \frac{Cb^2}{4}\left[f\left(\frac{L}{R_e}\right) + (IGS)_{CMWP}\right] .$$

The intergranular strains, ε_{IGS}, are obtained as:

$$\varepsilon_{IGS}(hkl) = b\sqrt{C \qquad (IGS)_{CMWP}} ,$$

where b is the Burgers vector, ρ^* is the dislocation density as given by CMWP and $(IGS)_{CMWP}$ is the number in eq. (3) given by CMWP. The *hkl* dependence of IGS is given by the contrast factors, *C*(*hkl*). If IGS is blank or zero no IGS is taken into account. IGS is fitted if IGS<0. IGS is taken into account but not fitted if IGS>0.

Number of phases:
There is the option to evaluate more than one phase simultaneously. In such a case here you have to put in the number of phases.

Select phase to edit:
When more than one phase is evaluated the second or more phase initial values have to be edited separately.

Call MKSpline, Call MKSpline2,
These activate the background spline adjustment. For more details see Ribárik et al. (2020).

Call MKSpline2
Activating this button shows the measured, the fitted and the background patterns in green, red and blue, respectively. Using the left-button on the mouse allows adjusting the background-spline (BG-Spl) fixpoints in order to bring the measured and calculated patterns closer to each other. This option allows subtle corrections of the background. Note that the default intensity scale is logarithmic, meaning that the lower intensity values and the background itself are strongly enlarged compared to the rest of the pattern. The BG-Spl fixpoints are also automatically vertically adjusted at the end of each fitting procedure.

Index peaks
This activates an auxiliary program for indexing and positioning the peaks producing the "peak_index.dat" file. Without this file the procedure does not start, see also the *phd-work of Ribarik*. The "peak_index.dat" file can be editied separately by any text editor and added to the initial files before starting CMWP.

Set individ. C values:
This activates an auxiliary JAVA frontend where the individual dislocation contrast factor values can be edited.

Clone INI files:
When starting to evaluate a new pattern which has similar initial values as a pattern already evaluated, the old "initial" values are implemented by using this option.

Save INI files:
This saves the initial values used in the evaluation.

Update peak-index.dat
This updates peak-index and peak-intensity values refined during the optimisation process.

(Re)Start FIT:
With this the evaluation procedure can start or re-start.

Stop FIT:
The evaluation procedure can be stopped/halted when pressing here. A "promt window" will appear showing the progress of stoppingthe actual "gnuplot" procedure. When Monte-Carlo (MC) and Levenberg-Marquardt (LM) procedures work together, see Ribárik et al. 2018; 2020, optimisation runs in six consequtive steps: (1) MC fitting only physical parameters, (2) LM fitting only peak intensities, (3) LM fitting only peak positions, (4) LM fitting only physical parameters, (5) LM fitting all parameters and (6) LM fitting only BG-Spl fixpoints. In this case all six steps have to be stoped subsequantly. If, however, only LM runs, all parameters are fitted together in a single step.

Update params:
This updates the parameter values when the total optimisation procedure has been completed.

View solutions:
This activated a "promt window" where the results and details of the optimisation procedure are shown.

Exit:
With this the evaluation procedure can be left.

Set fit parameters (Fig. 2 in "S5-JAVA-Frontend images")

This activates a pop-up window where the technicalities of the optimisation procedure can be edited.

min- , max-****
The first 9 rows allow to set hard limits of the minimum and maximum values of the physical parameters. It is advisable to allow wide ranges.

Use MC; Use ML
The usage of MC and ML (LM) procedures can be edited here. When both are allowed the six steps are active as described in detail in Ribárik et al. 2020.

****fit limit:**
The evaluation procedure is controlled by the weighted-sum-of-squared-residuals (WSSR), when the relative decrease of the WSSR reaches the value of "fit limit" the actual procedure will stop automatically; the default value is 10^{-9}.

The CMWP procedure provides the **weighted sum of squared residual** (WSSR) and the **goodness of fit** (GoF) at the end of the fitting procedure listed in the *.sol file.

Most of the editable paramters have self-explanatory names. More details about the organization of running CMWP are described in Ribárik et al. 2020.

References

Balogh, L., Ribárik, G., Ungár, T. (2006). *J. Appl. Phys*., **100**, 023512-10.

Balogh, L., Tichy, G., Ungár, T. (2009). *J. Appl. Cryst*., **42**, 580-591.

Balogh, L., Brown, D.W., Mosbrucker, P., Long, F. & Daymond, M.R. (2012). *Acta Mater*., **60**. 5567–5577.

Balogh, L., Long, F. & Daymond, M.R. (2016). *J. Appl. Cryst*. **49**, 2184-2200.

Balogh, L., Figueiredo, R. B., Ungár, T. & Langdon, T. G. (2010) *J. Mater. Sci. Eng. A,* **528**, 533-538.

Borbély, A., Dragomir-Cernatescu, J., Ribárik & Ungár, T. (2003). *J. Appl. Cryst*. **36**, 160-162.

Cordier, P., Ungár, T., Zsoldos, L. & Tichy, G. (2004) *Nature* **428**, 837-839.

Dragomir, I. C. & Ungár, T. (2002) *J. Powder Diffr.* **17**, 104-111.

Dragomir, I. C. & Ungár, T. (2002a). *J. Appl. Cryst*., **35**, 556-564.

Ribárik, G. (2008) in *Modeling of diffraction patterns based on microstructural properties, PhD Dissertation*, Eötvös University Budapest, Hungary.

Ribárik, G. & Ungár, T. (2010). *Mater. Sci. Eng*. **A528**, 112–121.

Ribárik, G., Jóni, B. & Ungár, T. (2019) *J. Mater. Sci. Technol*. **35**, 1509-1514.

Ribárik, G., Jóni, B. & Ungár, T. (2020) *Crystals*, 2020, 10, 623; doi:10.3390/cryst10070623

Ungár, T., Dragomir, I., Révész, Á. & Borbély, A. (1999) *J. Appl. Cryst*. **32**, 992-1002.

Ungár, T. & Tichy, G. (1999). *Phys. Stat. Sol*. **a171**, 425-434.

Ungár, T, Gubicza, J., Ribárik, G., Borbély, A. (2001). *J. Appl. Cryst*., **34**, 298-310.

Ungár, T, Ribárik, G., Balogh, L., Salem, A. A., Semiatin, S. L. & Vaughan, G. B. M. (2010) **63**, 69-72.

Comment

CMWP produces a fairly large number of output files, the two most useful for the user are the *.sol (the larger one) and the *.int4.dat files. The *.sol file consists of all input and output data and results corresponding to the last evaluation. The *.int4.dat file is a 4 column text file with the 2θ (or K) values, the measured, the calculated intensities and the difference values. All other output files contain useful information and can be tested by the user.
