# Supplementary material for: Characterizing dislocation loops in irradiated polycrystalline Zr alloys by X-ray line profile analysis of powder diffraction patterns with satellites
Source: J Appl Crystallogr. 2021 May 25;54(Pt 3):803–21. doi: 10.1107/S1600576721002673 (PMC8202032; doi:10.1107/S1600576721002673)
Supplement: Supplementary file 3 [file j-54-00803-sup3.pdf]

Supplement S3

Manual of the JAVA frontend of satellite evaluation in CMWP

2020

Gábor Ribárik and Tamás Ungár

This brief manual describes the pop-up window of "Set Satellite parameters" in the JAVA–frontend for the usage of satellite fitting. The best strategy is to go through **Supplement S1** first.

Satellite fitting can only be done in K-space. As a first step the pattern has to be optimized without satellites, i.e. "Use satellites:" should be inactive.

In the next step the evaluation has to be terminated and the script "convert-to-K" has to be applied. This step will convert the entire evaluation file system, related to this particular pattern, from 2θ to K. Note that if the original evaluation was done in K-space then this step must not be done. Once the file system is in K-space the "Use satellites:" can be activated.

In the next step the satellite initial parameter values can be edited in the "Set satellite parameters" pop-up window, see Fig. 3 in the Supplements S5 "JAVA-Frontend-images".

There is the option to fit four satellites.
The satellite parameters are:
S*i*_I: the global intensity of satellites relative to the peak-intensities of the main peaks,
S*i*_wl: the global median, m_{sat}, of the log-normal size distribution in the satellite profile function,
S*i*_η: the global variance, σ_{sat}, of the log-normal size distribution in the satellite profile function,
S*i*_s0: the global shift parameter of satellites,
S*i*_fit: this is the number controlling the satellite optimization procedure:

- S*i*_fit: "0" this satellite is not optimized, i.e. the parameter values remain unchanged during the optimization procedure
- S*i*_fit: "1" the satellite intensities are refined on top of the global intensity value S*i*_I,
- S*i*_fit: "2" only the global parameter values are refined,
- S*i*_fit: "3" only S*i*_wl, S*i*_η and S*i*_s0 are refined
- S*i*_fit: "4" all global parameters and the satellite intensities are refined

"*i*" is 1 to 4,

S*i*_a1: this is the "a_1" parameter in the *hcp* contrast factor of strained objects generating satellites
S*i*_a2: this is the "a_2" parameter in the *hcp* contrast factor of strained objects generating satellites

Note that the satellite option has been developed primarily for dislocation loops generating satellites in irradiated *hcp* polycrystalline materials. Therefore, the contrast factor parameters in Fig. 3 in the Supplements S5 "JAVA-Frontend-images" are of dislocation loops in neutron irradiated Zr.

The signs of the s0 global shift parameters are hard-coded:

"*i*"=1: s0<0, i.e. this satellite is on the smaller 2θ side of the main peak

"*i*"=2: s0>0, i.e. this satellite is on the larger 2θ side of the main peak

"*i*"=3: s0<0, i.e. this satellite is on the smaller 2θ side of the main peak

"*i*"=4: s0>0, i.e. this satellite is on the larger 2θ side of the main peak

Important: when a satellite is not used, as the second and third in the front-end figure, the global intensity value should be set to a small but not zero value.

Satellite optimization is done by the same six-step MC plus LM (ML) procedure as the main pattern optimization. Satellite and main pattern optimization is done separately, i.e. when one is done the other is unchanged. Therefore, it is advisable to carry out satellite and main pattern optimizations consecutively a few times until convergence is reached.
