# Supplementary material for: Characterizing dislocation loops in irradiated polycrystalline Zr alloys by X-ray line profile analysis of powder diffraction patterns with satellites
Source: J Appl Crystallogr. 2021 May 25;54(Pt 3):803–21. doi: 10.1107/S1600576721002673 (PMC8202032; doi:10.1107/S1600576721002673)

Figure 1.
Java frontend

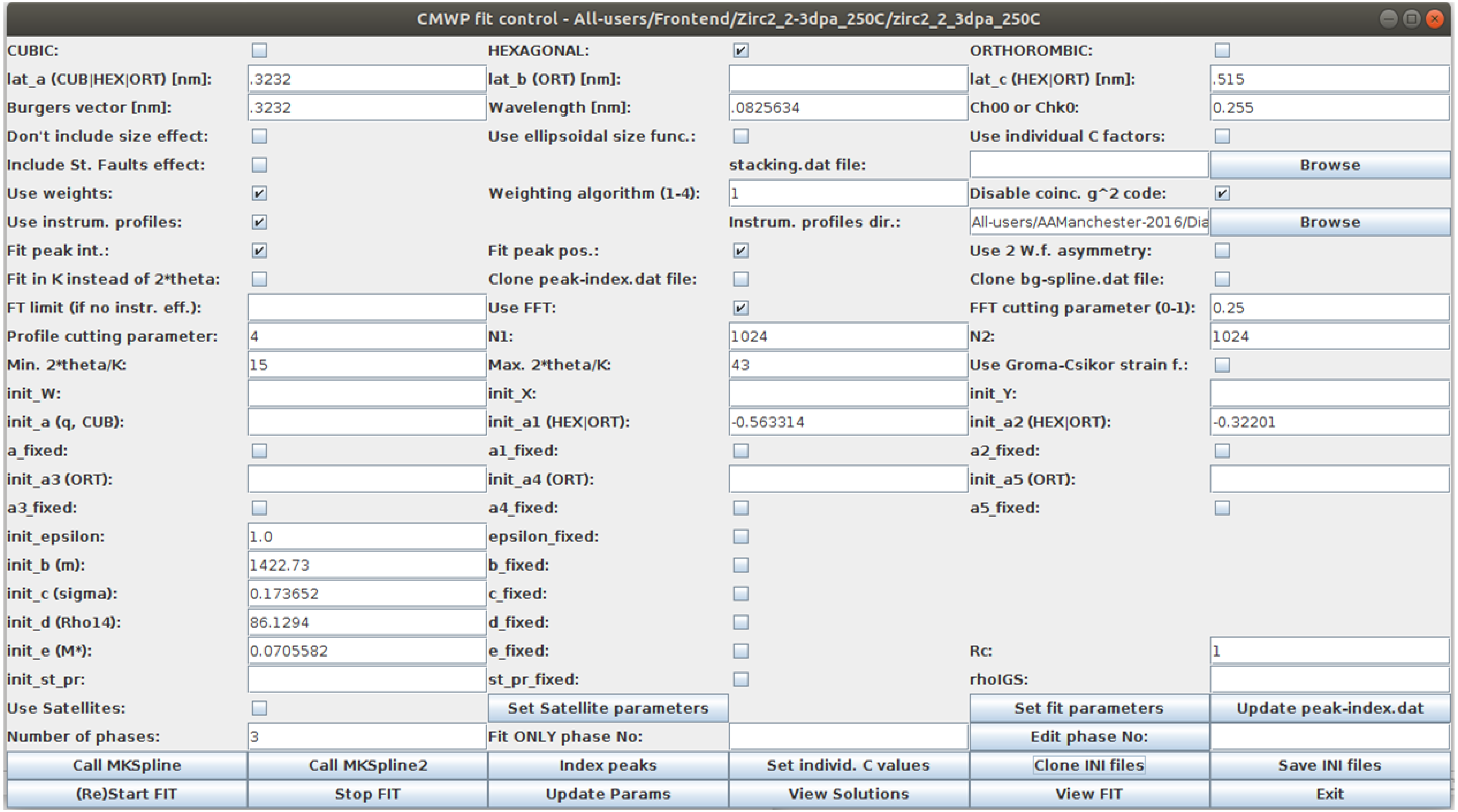

Figure 2.
"Set fit parameters" pop-up window

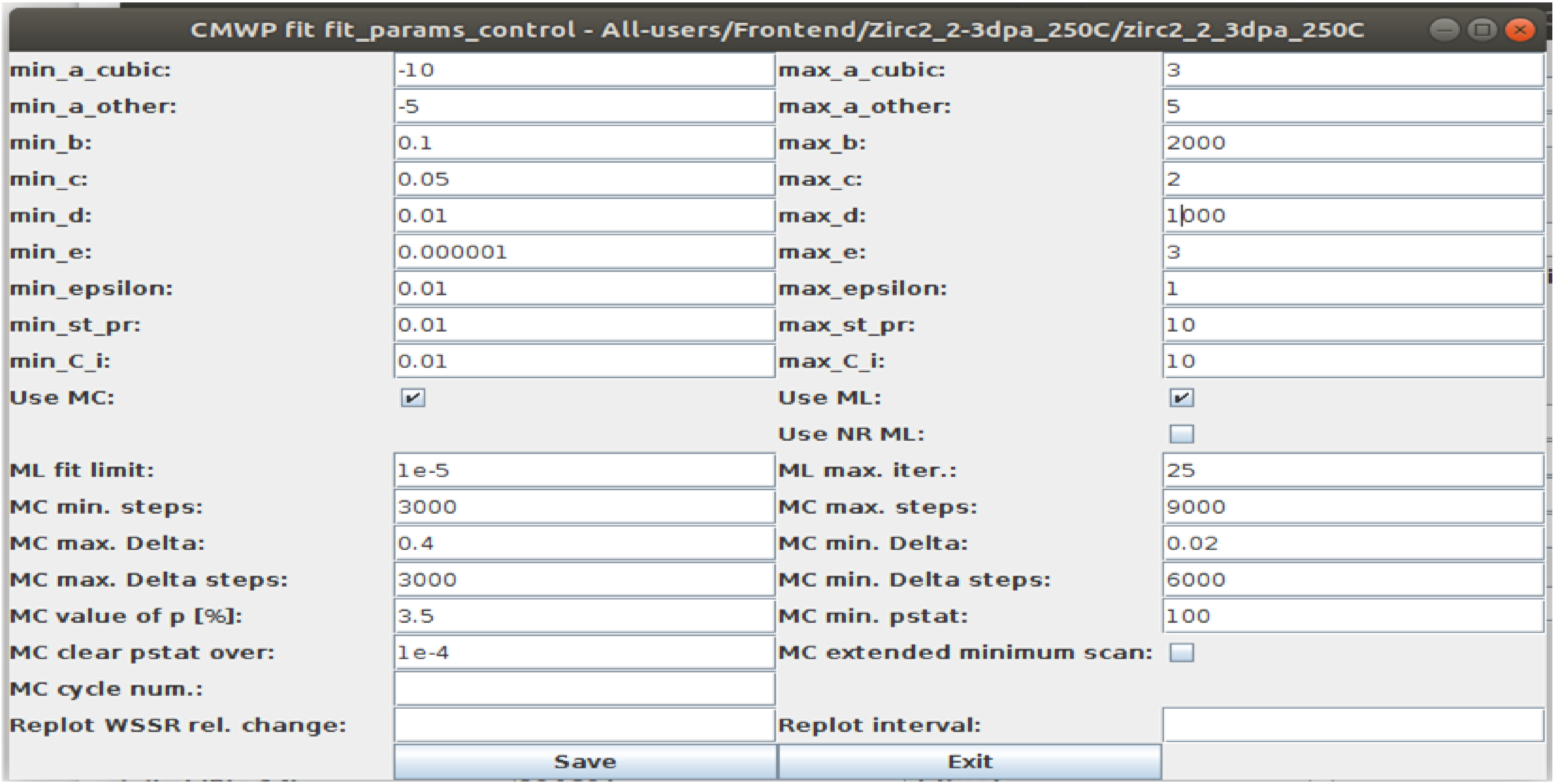

Figure 3.
"Set satellite parameters" pop-up-window

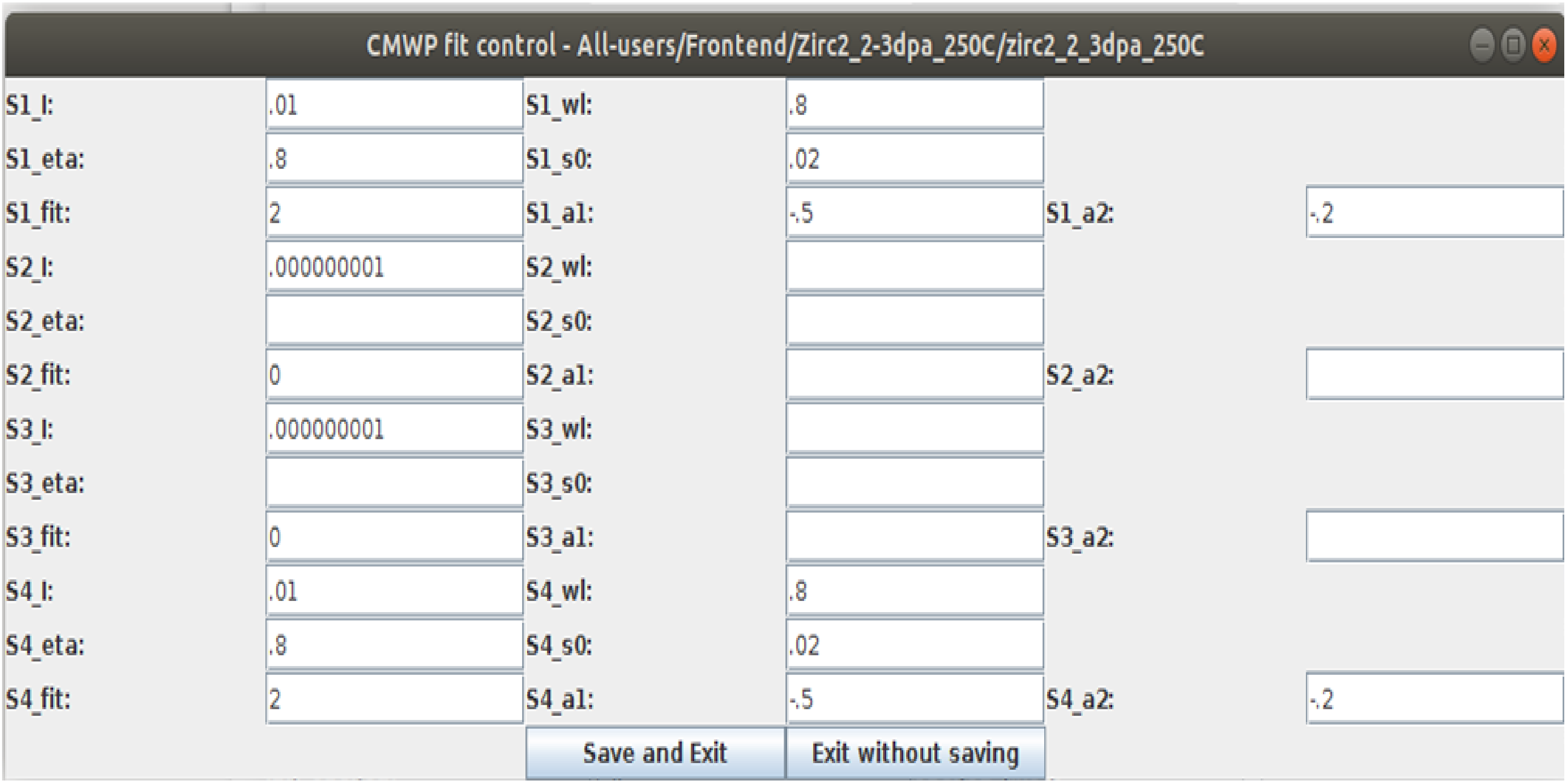

Figure 4.
"Call MKSpline" pop-up window

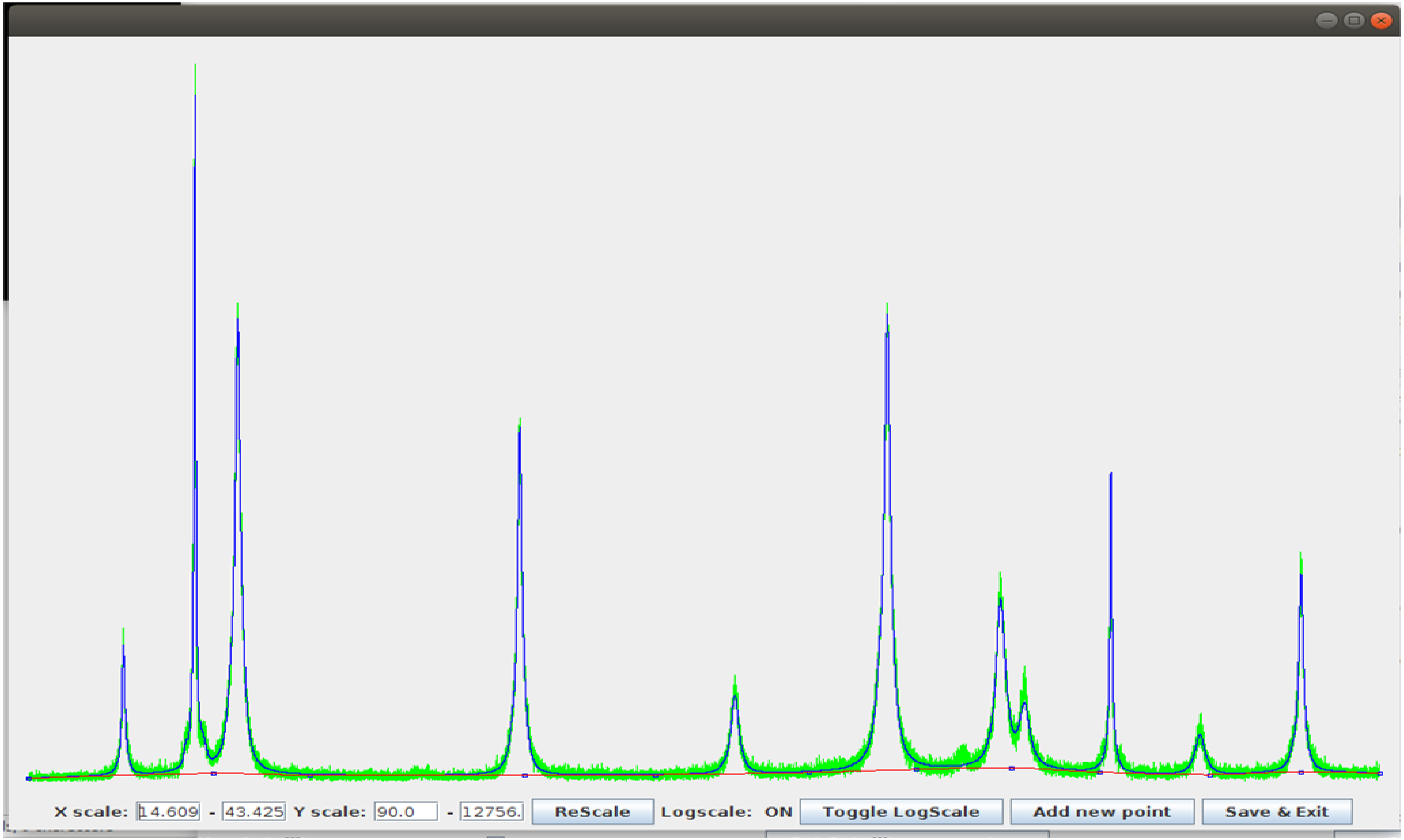

Figure 5.
Zoomed intensity scale of "Call MKSpline" pop-up window

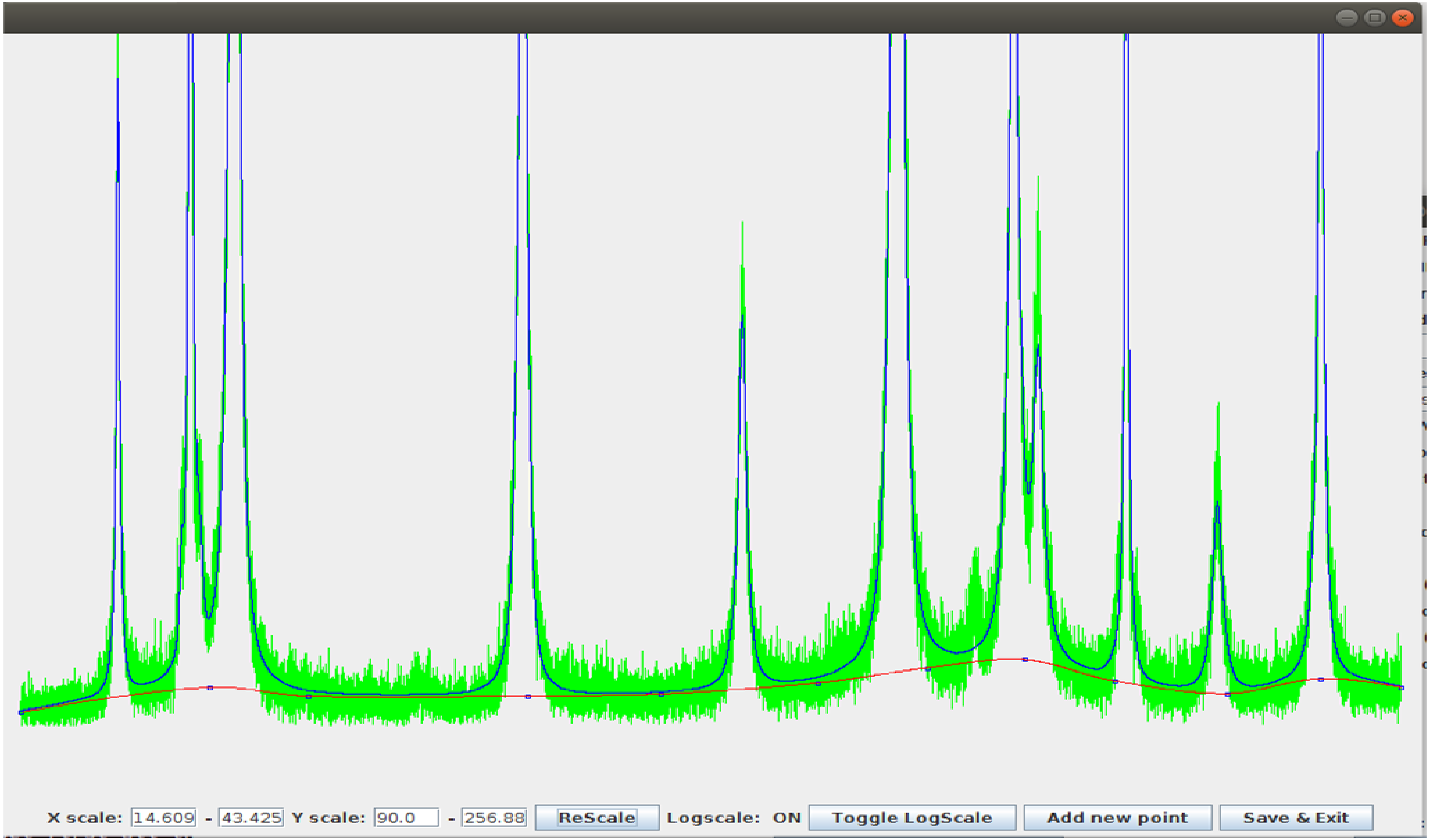

Figure 6.
Zoomed horizontal scale of "Call MKSpline" pop-up window

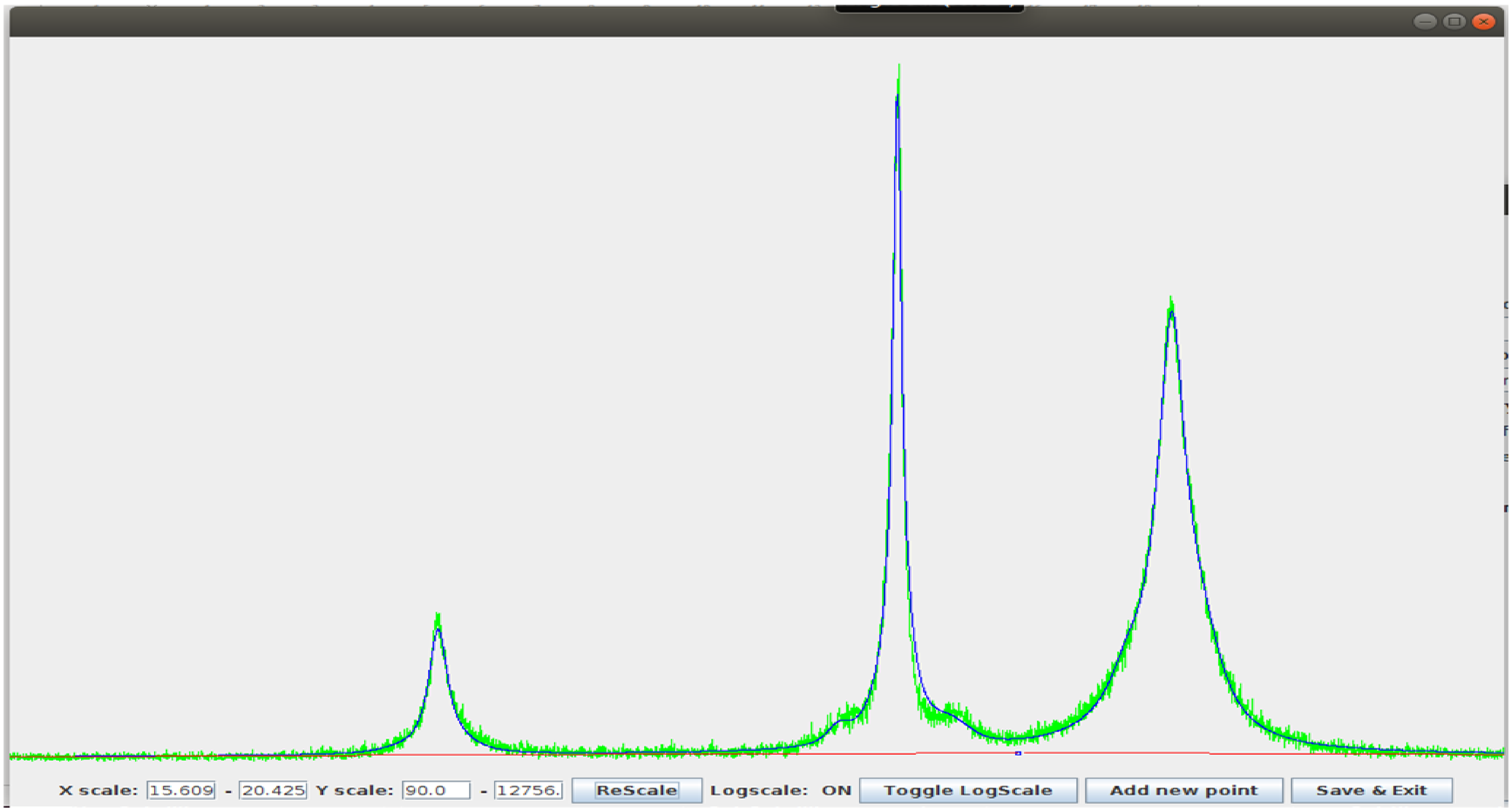

Figure 7.
Gnuplot image of fitting along with satellites (dash blue lines) available from the output files once the fitting procedure was done. (Note that the satellite peaks are added to the background spline.)

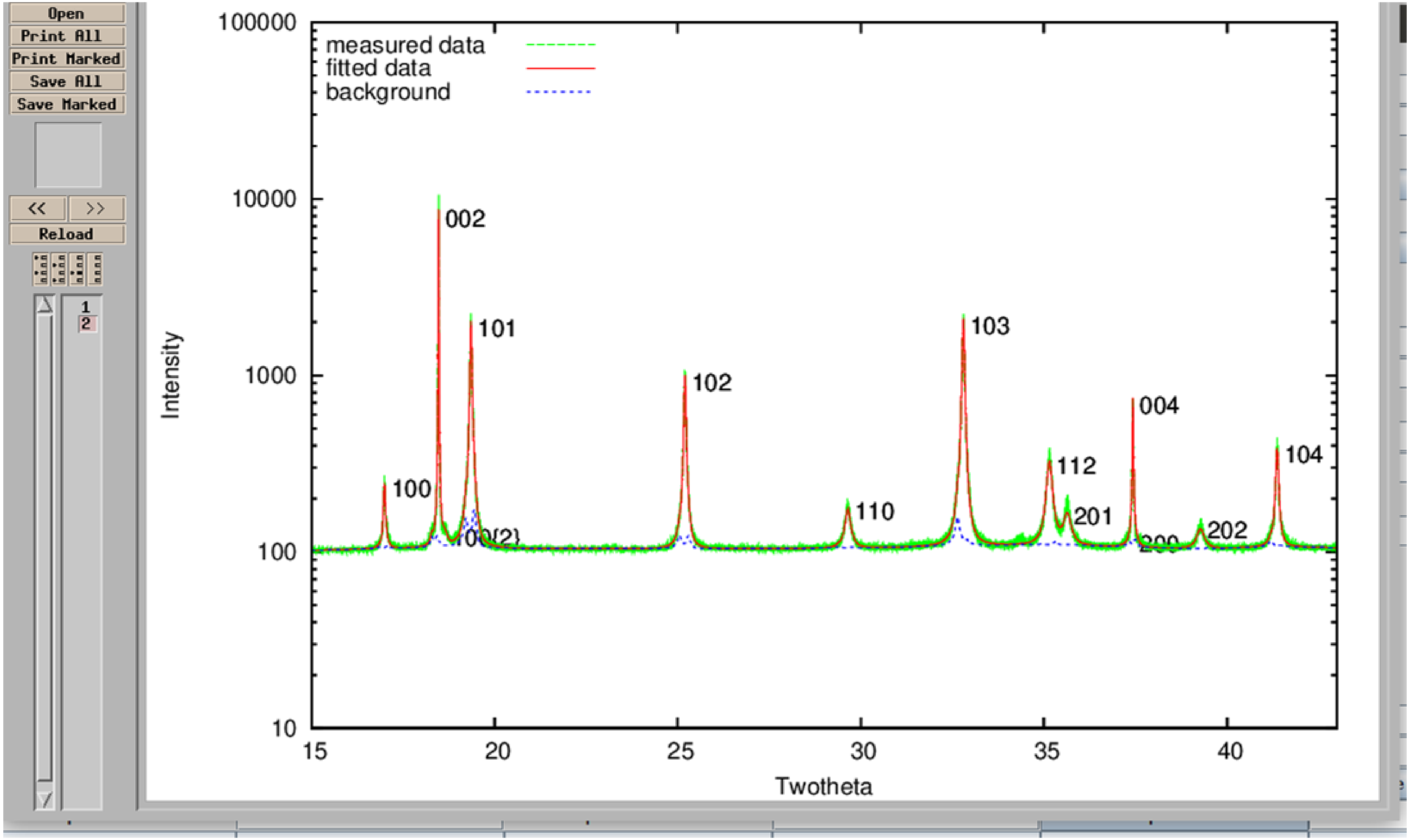

Supplement: Supplementary file 4 [file j-54-00803-sup4.pdf]
